# Formation of colloidal alloy semiconductor CdTeSe magic-size clusters at room temperature

**DOI:** 10.1038/s41467-019-09705-w

**Published:** 2019-04-11

**Authors:** Dong Gao, Xiaoyu Hao, Nelson Rowell, Theo Kreouzis, David J. Lockwood, Shuo Han, Hongsong Fan, Hai Zhang, Chunchun Zhang, Yingnan Jiang, Jianrong Zeng, Meng Zhang, Kui Yu

**Affiliations:** 1 Institute of Atomic and Molecular Physics, Sichuan University, 610065 Chengdu, P. R. China; 2 Metrology Research Centre, National Research Council of Canada, Ottawa, ON K1A 0R6 Canada; 3 School of Physics and Astronomy, Queen Mary University of London, London, E1 4NS UK; 4 School of Physical Science and Technology, Sichuan University, 610065 Chengdu, P. R. China; 5 Engineering Research Center in Biomaterials, Sichuan University, 610065 Chengdu, P. R. China; 6 Analytical & Testing Center, Sichuan University, 610065 Chengdu, P. R. China; 7 Jilin Ginseng Academy, Changchun University of Chinese Medicine, 130117 Changchun, P. R. China; 8 Shanghai Synchrotron Radiation Facility, Shanghai Advanced Research Institute, Chinese Academy of Sciences, 201204 Shanghai, P. R. China; 9 Shanghai Institute of Applied Physics, Chinese Academy of Sciences, 201800 Shanghai, P. R. China; 10 State Key Laboratory of Polymer Materials Engineering, Sichuan University, 610065 Chengdu, P. R. China

## Abstract

Alloy semiconductor magic-size clusters (MSCs) have received scant attention and little is known about their formation pathway. Here, we report the synthesis of alloy CdTeSe MSC-399 (exhibiting sharp absorption peaking at 399 nm) at room temperature, together with an explanation of its formation pathway. The evolution of MSC-399 at room temperature is detected when two prenucleation-stage samples of binary CdTe and CdSe are mixed, which are transparent in optical absorption. For a reaction consisting of Cd, Te, and Se precursors, no MSC-399 is observed. Synchrotron-based in-situ small angle X-ray scattering (SAXS) suggests that the sizes of the two samples and their mixture are similar. We argue that substitution reactions take place after the two binary samples are mixed, which result in the formation of MSC-399 from its precursor compound (PC-399). The present study provides a room-temperature avenue to engineering alloy MSCs and an in-depth understanding of their probable formation pathway.

## Abstract (web summary)

Alloy magic-size clusters (MSCs) are difficult to synthesize, in part because so little is known about how they form. Here, the authors produce single-ensemble alloy CdTeSe MSCs at room temperature by mixing prenucleation-stage solutions of CdTe and CdSe, uncovering a formation pathway that may extend to the synthesis of other alloy MSCs.

## Introduction

Colloidal alloy semiconductor magic-size clusters (MSCs) have been reported only in a very limited fashion^1,2^, and their syntheses have been acknowledged to be quite challenging via conventional hot-injection and heating-up approaches^3–5^. For the synthesis of metal (M) chalcogenide (E) semiconductor alloys, such as ZnCdSe^1^ and CdTeSe^2^ MSCs and CdTeSe quantum dots (QDs)^6,7^, corresponding M and E precursors are usually placed together in a reaction flask. The optical absorption reported of the ZnCdSe and CdTeSe MSCs seems to consist of two electronic transitions in the range of 300 to 350 nm for the former^1^, and at 464 and 520 nm for the latter^2^.

With relatively uniform size distributions, MSCs exhibit relatively narrow optical absorption, compared to corresponding QDs^8–15^. Very recently, a two pathway model has been proposed to be available in the prenucleation stage^16^, which is also called the induction period (occurring prior to nucleation and growth of QDs)^17^. It is suggested that one pathway follows the LaMer model of the classical nucleation theory (CNT)^18–20^, which involves monomers and fragments for which M-E covalent bonds form. The other pathway is argued to start with the self-assembly of M and E precursors followed by the formation of M-E covalent bonds inside the assembled species, each of which results in a special precursor compound (PC) of MSCs^21^. The PC (in a conventional solvent such as toluene or hexane or cyclohexane) is transparent in optical absorption, and has the character that one PC molecule can transform into a corresponding MSC (following first-order reaction kinetics)^22,23^. The two pathways are linked by a MSC → PC transformation and PC fragmentation to QDs^16^. Usually, PCs form before monomers and fragments do (when the concentrations of M and E precursors in a reaction are not too low)^21^.

The two pathway model indicates the distinct possibility of a selective two-step approach to the exclusive production of MSCs in a single ensemble form, at the same time without the complication of the presence of QDs^24^. The first step is to produce the PC at a relatively high temperature but still within the induction period. The second step is to form MSCs at a lower temperature, such as room temperature, from the PCs produced in an induction period sample. This two-step method has some definite and obvious differences to the conventional hot-injection and heating-up approaches^3–5^, and has been validated to be efficient in producing binary CdTe MSC-371^23,24^, CdSe MSC-415^25^, ZnSe MSC-299^21^, CdS MSC-311^16,22,26^, and MSC-322^26^. These MSCs have distinctive sharp optical absorption peaks at the wavelength of ~371, 415, 299, 311, and 322 nm, respectively. However, there has been no report on alloy ternary MSCs via the selective two-step approach, and the formation pathway of alloy MSCs remains largely unexplored.

Chalcogenide (E) anion exchange reactions have been reported for semiconductor nanoparticles (NPs)^27–31^, so have cation exchange reactions^32–36^. Such chemical transformations have been accepted to be promising alternatives to producing colloidal NPs with improved control over composition and morphology. For example, E-based partial anion exchange reactions have been reported, which allow the preparation of CdS/CdTe heterodimers from CdS (with tri-*n*-octylphosphine telluride (Te=P(C_8_H_17_)_3_, TeTOP at 260 °C)^27^, and ternary CdTeS NPs from CdTe (with Na_2_S at 40 °C in water)^28^. Also, anion exchange reactions (with TeTOP at the temperature range of 220–300 °C) have been shown to be effective for converting selenides to tellurides^29^. For the anion exchange reactions with TeTOP at high temperatures^27,29^, the chemical driving force was attributed to the fact that the Te=P bond (~218 kJ mol^−1^) is weaker than the S=P (~402 kJ mol^−1^) and Se=P (~314 kJ mol^−1^) bonds (based on tri-*n*-butylphosphine chalcogenide instead of ETOP)^37^. For the anion exchange reactions in water^28^, they were reasoned to be influenced by the fact that the binding energy (~92 kJ mol^−1^) of the Cd–Te bond is smaller than that (~157 kJ mol^−1^) of the Cd–S bond^38^.

**Fig. 1:**
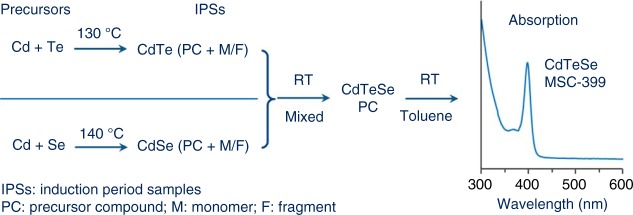
Schematic outlining the synthesis of alloy CdTeSe MSC-399 and formation hypothesis. The alloy MSCs are prepared at room temperature from the mixture of the two induction period samples (IPSs) of binary CdTe and CdSe (Equation (1)). Cd and Te or Cd and Se precursors are independently mixed at room temperature and heated at ~130 or ~140 °C to prepare the IPS, which has the precursor compounds (PCs) of CdTe MSC-371 or CdSe MSC-415, respectively, together with corresponding monomers (M) and fragments (F). The binary samples are mixed at room temperature for a period of time to produce MSC-399 via its precursor compound (CdTeSe PC, (Equation (4)), which is proposed to form via substitution reactions of the CdTe PC with the CdSe monomers and fragments (Equation (2)), and of the CdSe PC with the CdTe monomers and fragments (Equation (3))

Here, we report the synthesis of colloidal alloy CdTeSe MSC-399 and describe our argument for the formation pathway, as illustrated in Fig. 1. The MSCs obtained exhibit a single persistent and sharp absorption singlet peaking at 399 nm. Separately, two induction period samples of binary CdTe and CdSe are prepared at relatively high temperatures (120–140 °C)^22–24^; they are mixed afterwards at room temperature. The resulting mixture, together with the two binary samples, are characterized by optical absorption spectroscopy, electrospray ionization mass spectrometry (ESI-MS), and ^113^Cd nuclear magnetic resonance (NMR). Evidently, alloy CdTeSe MSC-399 in a single ensemble form evolves from the mixture, without the emergence of other-bandgap MSCs and QDs. To explore the formation pathway of MSC-399, some control experiments were carried out, together with synchrotron-based in situ small angle X-ray scattering (SAXS). Critically, SAXS suggests that the two binary samples and their mixture all have similar particle sizes. For the formation of CdTeSe MSC-399 from the two binary induction period samples (IPS) (Equation (1)), we propose that it is the substitution reactions (Equations (2) and (3)), predominating over the addition reaction of the CdTe and CdSe precursor compounds (PCs), that result in the production of the precursor compound (CdTeSe PC) for CdTeSe MSC-399 (Equation (4)).

1 $${\mathrm{CdTe}}\,{\mathrm{IPS}} + {\mathrm{CdSe}}\,{\mathrm{IPS}} \to {\mathrm{CdTeSe}}\,{\mathrm{MSC - 399}}$$

2 $${\mathrm{CdTe}}\,{\mathrm{PC}} + {\mathrm{CdSe}}\,{\mathrm{M/F}} \to {\mathrm{CdTeSe}}\,{\mathrm{PC}}$$

3 $${\mathrm{CdSe}}\,{\mathrm{PC}} + {\mathrm{CdTe}}\,{\mathrm{M/F}} \to {\mathrm{CdTeSe}}\,{\mathrm{PC}}$$

4 $${\mathrm{CdTeSe}}\,{\mathrm{PC}} \to {\mathrm{CdTeSe}}\,{\mathrm{MSC - 399}}$$

CdTe PC and CdSe PC represent the molecules that are able to transform to CdTe MSC-371 and CdSe MSC-415, respectively. CdTe M/F and CdSe M/F are symbolic of the binary monomers/fragments, which are produced in the two corresponding binary CdTe IPS and CdSe IPS, respectively. The present study introduces a room-temperature approach to producing alloy MSCs in a single ensemble form without the co-production of other-bandgap MSCs and QDs, and provides insight into their probable formation pathways.

## Results

### Evolution of alloy CdTeSe MSC-399

**Fig. 2:**
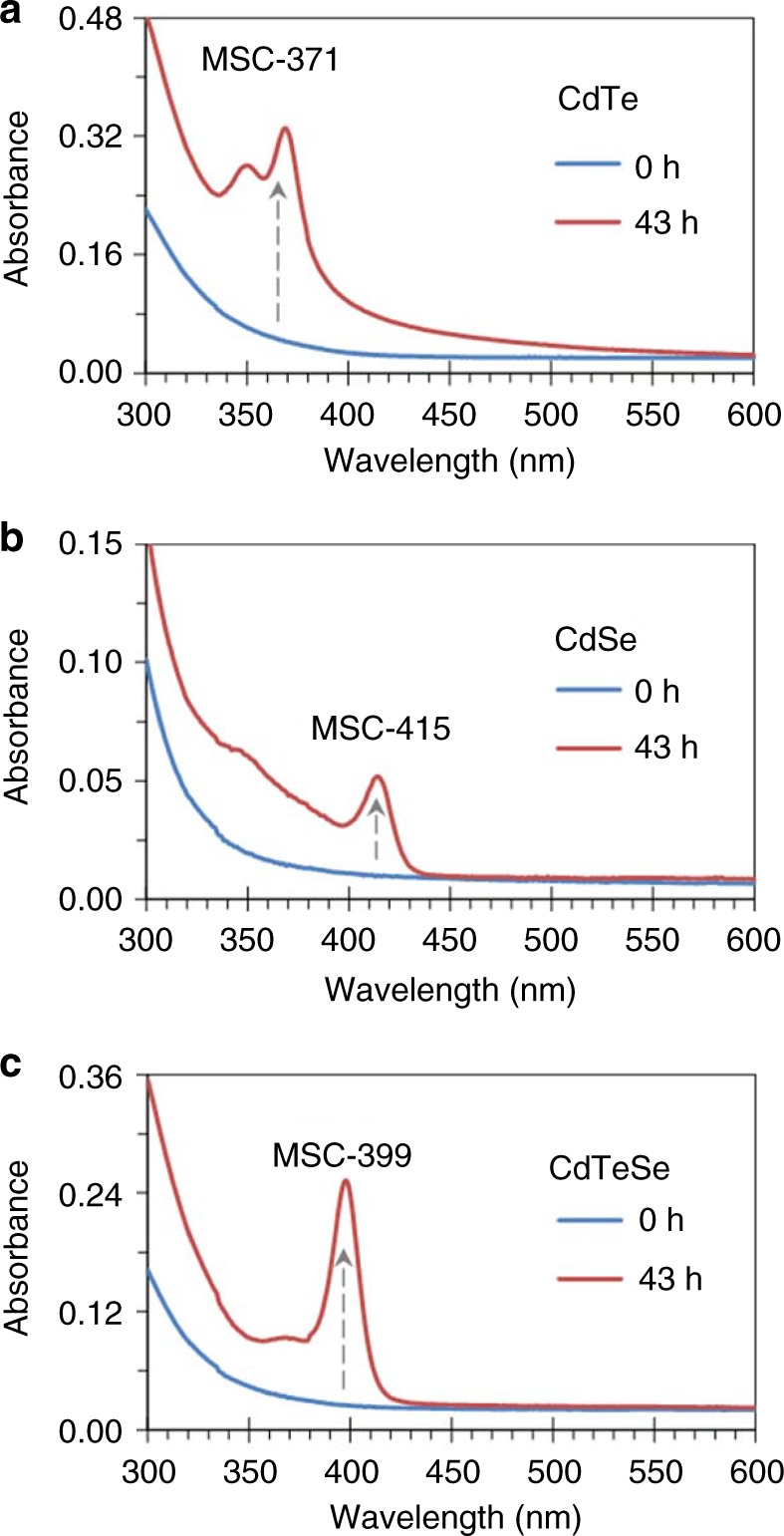
Evolution of binary and ternary alloy MSCs. Optical absorption spectra of the induction period samples of binary CdTe (**a**) and CdSe (**b**), and their mixture (**c**) are collected before (blue traces) and after a 43-h incubation (red traces) at room temperature. The CdTe and CdSe samples were heated at 130 and 140 °C for 30 min, respectively; they were cooled to room temperature and mixed with equal volumes. An aliquot of each sample (30 μL) was dispersed in toluene (3.0 mL) for the measurement. During incubation, evidently, CdTe MSC-371 and CdSe MSC-415 evolved from the corresponding binary samples, while only CdTeSe MSC-399 evolved from their mixture

Figure 2 shows the optical absorption spectra of two induction period samples of binary CdTe (a) and CdSe (b), together with their mixture (c) at room temperature before (blue traces) and after 43-h incubation (red traces). The CdTe sample (a) was obtained after the Cd and Te precursors were mixed at room temperature and heated at 130 °C for 30 min^23,24^, while the Cd and Se precursors were mixed at room temperature and heated at 140 °C for 30 min to produce the CdSe sample (b)^25^. The two binary samples were mixed in equal volumes at room temperature (c). An aliquot (30 μL) of each of the three samples before and after incubation was dispersed in toluene (3.0 mL), and its absorption spectrum was collected.

The blue traces illustrate that the binary samples and their mixture are transparent in optical absorption. The three featureless spectra suggest the absence of MSCs in these samples. The red traces of the incubated samples, exhibiting characteristically narrow absorption peaks, demonstrate the presence of CdTe MSC-371 (a), CdSe MSC-415 (b), and probable CdTeSe MSC-399 (c). The presence of the binary MSCs indicates the presence of their corresponding precursor compounds (PCs) in the two binary samples, together with monomers and fragments, and that the CdTe PC → MSC-371 and CdSe PC → MSC-415 transformations took place during incubation at room temperature^16,21–26^.

For the mixture of the binary samples after incubation, only the 399 nm peak is observed (red trace in Fig. 2c). Accordingly, we attribute it to a ternary CdTeSe system and label it MSC-399. The presence of MSCs is further supported by the persistent peak position at 399 nm as seen in Supplementary Figs. 1 to 3. Importantly, neither CdTe MSC-371 nor CdSe MSC-415 is present in the incubated mixture. Accordingly, during the mixture incubation, the CdTe PC → MSC-371 and CdSe PC → MSC-415 transformations apparently do not occur^16,23,25^, in accordance with the relevant process of the substitution reactions described by Equations (2) and (3), respectively. Hence, we conclude that during the mixture incubation, the reactions indicated by Equations (2) to (4) take place probably.

For the synthesis of MSC-399, we explore the effects of the temperatures used when heating the two binary CdTe and CdSe samples (Supplementary Fig. 1), together with the heating periods (Supplementary Fig. 2) and their mixing volume ratios (Supplementary Fig. 3). The substitution reactions described by Equations (2) and (3) are able to explain well the experimental results obtained. Remarkably, for the samples extracted from the reaction batch consisting of the Cd, Te, and Se precursors together (such as during the temperature range of 120–150 °C), apparently, no MSC-399 is present (Supplementary Fig. 4). We argue that this absence is due to the fact that the formation of Cd–Te bonds takes place at a lower temperature than that needed for Cd–Se bonds (in a reaction batch)^2,6,7,23–25^. Furthermore, the formation of CdTe QDs (taking place at ~150 °C (trace 4 in Supplementary Fig. 4)) consumed the CdTe PC produced (via the PC fragmentation to QD pathway)^15^. Thus, the formation of CdTeSe PC was hindered.

### ESI-MS of binary samples and their mixture

ESI-MS has been applied to investigate the formation of M-E covalent bonds in the first-step sample of the selective two-step approach; the first step is controlled to be within the induction period^21,24,25^. For those fragments detected, they do not seem to display any surface ligands. Furthermore, the fragment interval between two nearest-neighbor peaks is 1 Da, indicating that the bare fragments are mono-charged^39,40^.

**Fig. 3:**
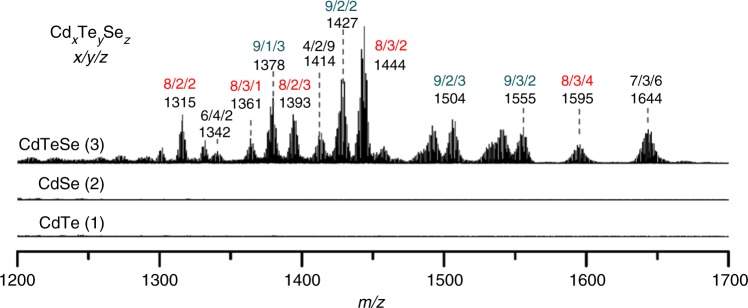
ESI-MS spectra of two binary samples and their mixture. Both the binary CdTe (trace 1) and CdSe (trace 2) samples were heated at 120 °C for 30 min; they were mixed at room temperature with equal volumes; the resulting mixture (trace 3) was incubated for 30 min. The Cd_*x*_Te_*y*_Se_*z*_ fragments (trace 3) suggest the formation of Te–Cd-Se covalent bonds in the mixture, which is in agreement with the substitution reactions described by Equations (2) and (3)

Figure 3 displays the ESI-MS spectra within the *m/z* range of 1200–1700 Da, for the samples of binary CdTe (trace 1) and CdSe (trace 2) (without room temperature incubation), together with their room temperature mixture (trace 3) with equal volumes and 30 min incubation. The CdTe and CdSe samples were heated at 120 °C for 30 min. Supplementary Fig. 7 presents the same ESI-MS collection but in the *m/z* range of 950–1250 Da. For the fragments detected from the mixture (trace 3), some of them are unambiguously different from those monitored from the two binary samples (traces 1 and 2). We tried to assign these fragments to Cd_*x*_Te_*y*_Se_*z*_ (where x, y, and z are integer values), based on the Cd, Te, Se, Cd_*1*_Te_*1*_, Cd_*1*_Se_*1*_, and Cd_*1*_Te_*1*_Se_*1*_ isotopic patterns and peak positions (Supplementary Fig. 8 and Supplementary Note 1)^20,23,24^. Supplementary Fig. 9 presents the expanded views of the Cd_*x*_Te_*y*_Se_*z*_ fragments assigned in the *m/z* range of 950–1700 Da. Based on the formula of x + y + z (= 9–16), it seems that the two 11-atom species, Cd_*7*_Te_*1*_Se_*3*_ and Cd_*6*_Te_*2*_Se_*3*_, were produced most often, among the fragmented species consisting of 9–16 atoms. The formation of the mono-charged fragments could be related to surface ligand detachment;^21,24,25,41,42^ intriguingly, the charge of the fragments presented here is different from the overall charge calculated based on Cd^2+^, Te^2-^, and Se^2−^.

For the binary samples of CdTe 130 °C/30 min and CdSe 140 °C/30 min, together with their mixture used for Fig. 2, Supplementary Fig. 10 presents their ESI-MS spectra. Again, some of the fragmented species detected from the mixture differ from those from the two binary samples. Supplementary Fig. 11 highlights the comparison of the fragments obtained, for the CdTe 120 °C/30 min vs 130 °C/30 min samples, the CdSe 120 °C/30 min vs 140 °C/30 min samples, and their corresponding mixtures. It seems that, for the two binary samples, more fragments are obtained from the higher temperature samples. Accordingly, Fig. 3 and Supplementary Fig. 10 provide indirect but convincing evidence for the formation of Te–Cd–Se covalent bonds in the mixture of the two binary samples. The ESI-MS study is thus supportive of the relevance of the substitution reactions represented by Equations (2) and (3), and that the substitution reactions readily take place at room temperature (within 30 min). The ESI-MS study is in agreement with the statement that MSC-399 has a ternary nature.

### ^113^Cd NMR of binary samples and their mixture

In view of its sensitivity to the local environment, ^113^Cd NMR spectroscopy has been used to follow the formation of Cd–Te covalent bonds in induction period samples^24^. Also, it has provided important information regarding the formation of alloy CdTeSe MSC-520 and CdTeSe QDs^2,7^. We now present the NMR investigation of the formation of Te–Cd–Se covalent bonds in the mixture of two binary samples of CdTe and CdSe.

**Fig. 4:**
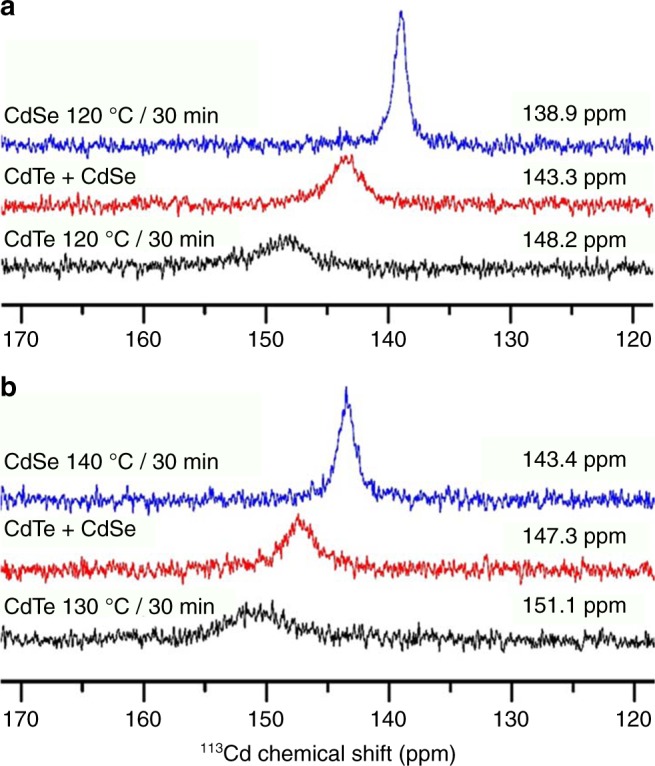
^113^Cd NMR spectra of two sets of binary samples and their mixtures. **a** Both binary samples for CdTe (black trace) and CdSe (blue trace) were reacted at 120 °C for 30 min, and the mixture (red trace) was obtained by mixing the two binary samples with equal volumes at room temperature for 15 min incubation. **b** CdTe (black trace) and CdSe (blue trace) samples were reacted for 30 min at 130 and 140 °C, respectively, and their mixture (red trace) was obtained with equal volumes at room temperature for 15 min incubation. The binary samples were prepared as indicated. The ^113^Cd resonance signals of the two mixtures (red traces), locating between those from the binary samples, are in agreement with the substitution reactions described by Equations (2) and (3)

Figure 4 shows the ^113^Cd NMR spectra collected from two CdTe samples (black traces), two CdSe samples (blue traces), and the corresponding two mixtures (red traces, with 15 min incubation at room temperature). Cd(ClO_4_)_2_ is used as a chemical shift reference. The binary samples in Fig. 4a are heated at 120 °C for 30 min, whereas the samples in the Fig. 4b are heated for 30 min at 130 °C for CdTe and at 140 °C for CdSe. Again, the two mixtures were prepared via the equal volume mixing of the two sets of the binary samples. The samples (0.3 mL) were diluted with toluene-*d*_*8*_ (0.3 mL). Supplementary Table 1 provides the details for the data collection.

For the two spectra displayed in red, the ^113^Cd resonance signals obtained are located between those of the corresponding binary samples. Accordingly, the two resonance signals are consistent with the formation of Te–Cd–Se covalent bonds via the substitution reactions denoted by Equations (2) and (3). Interestingly, the ^113^Cd resonance signals shift down-field, from the CdTe 120 °C sample (148.2 ppm) to 130 °C sample (151.1 ppm), and from the CdSe 120 °C sample (138.9 ppm) to 140 °C sample (143.4 ppm). Such shifts are attributed to the formation of more Cd–Te or Cd–Se bonds^24^. At the same time, there is a certain degree of down-field shifting for the ^113^Cd resonance signals from the mixture depicted in Fig. 4a (143.3 ppm) to the mixture of Fig. 4b (147.3 ppm). Accordingly, the concentration of Te–Cd–Se bonds in the Fig. 4a mixture appears to be smaller than that in the Fig. 4b mixture; this difference is consistent with the results shown in Supplementary Figs. 1 and 11. It is of help to point out that the NMR measurements are performed without sample purification, as we did for the CdTe binary system^24^. The presence of one single ^113^Cd resonance signal was argued to be related to fast chemical exchange processes in one sample^24^. We anticipate the same for the present study.

To further endorse our comprehension of the red trace shown in Fig. 4b, which is indicative of the formation of the alloy PC (from substitution reactions described by Equations (2) and (3)), we design a background experiment (Supplementary Fig. 12), in which two binary samples of CdTe and CdSe are placed closely enough but without mixing. Supplementary Fig. 12 illustrates the experimental arrangement and displays the NMR spectrum collected from the samples of CdTe 130 °C/30 min and CdSe 140 °C/30 min. This spectrum in Supplementary Fig. 12 has two discernible ^113^Cd resonance signals at ~151.8 and ~143.0 ppm and is understandably different from the corresponding spectrum (red trace) in Fig. 4b. The chemical shift values are similar to those of the CdTe (black trace) and CdSe (blue trace) spectra shown in Fig. 4b, respectively. This Supplementary Fig. 12 result provides further compelling evidence which supports the substitution reactions designated by Equations (2) and (3).

## Discussion

**Fig. 5:**
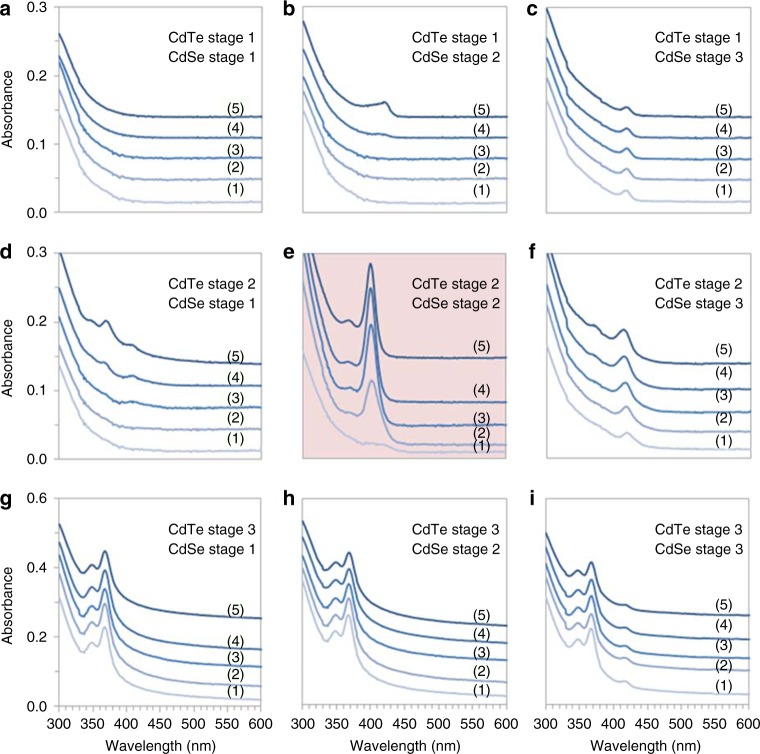
Optical absorption spectra of nine mixtures of CdTe and CdSe. There are nine mixtures prepared by mixing CdTe and CdSe samples in Stages 1 and 1 (**a**), 1 and 2 (**b**), 1 and 3 (**c**), 2 and 1 (**d**), 2 and 2 (**e**), 2 and 3 (**f**), 3 and 1 (**g**), 3 and 2 (**h**), and 3 and 3 (**i**), at room temperature with equal volumes. The mixtures were incubated for (1) 0 min, (2) 1 h, (3) 2 h, (4) 4 h, and (5) 8 h. Each of the mixtures (30 μL) was dispersed in toluene (3.0 mL). Interestingly, MSC-399 was well evolved only from the mixture shown in Part e with the mixture of Stage 2 CdTe and CdSe. Stage 1 means when two precursors of Cd and Te or Cd and Se were put together at room temperature; after Stage 1 samples were heated for 30 min at 130 °C for CdTe or at 140 °C for CdSe, they are referred as Stage 2 (induction period) samples. When the Stage 2 samples were incubated at room temperature for 24 h, they are called Stage 3 samples (with the presence of CdTe MSC-371 or CdSe MSC-415)

With regard to the formation pathway of MSC-399, we first considered chalcogenide (E) anion exchange reactions. Supplementary Fig. 13 shows the optical absorption properties of the mixture of CdTe (130 °C/30 min) and SeTOP, as well as those of the mixture of CdSe (140 °C/30 min) and TeTOP. For the former mixture, CdTe MSC-371 is monitored (after 4 h of mixing), while any possible CdSe MSC-415 is sought from the latter mixture. Also, it is apparent that no CdTeSe MSC-399 evolves from the two mixtures. Accordingly, it seems reasonable that anion exchange reactions (with SeTOP or TeTOP) do not take place at room temperature. In addition to Supplementary Fig. 13, additional control experimental results are presented in Fig. 5, which addresses the possible formation of MSC-399 from mixtures made from binary samples at three stages.

The CdTe and CdSe samples used are categorized as Stages 1–3. When the two precursors of Cd and Te or Cd and Se were put together at room temperature (in oleylamine (OLA) to prepare one induction period sample), the resulting mixture is referred to as a Stage 1 sample. After the Stage 1 sample was heated for 30 min at 130 °C for CdTe or at 140 °C for CdSe, it is designated as a Stage 2 sample. When Stage 2 samples were incubated at room temperature for 24 h (during which CdTe MSC-371 and CdSe MSC-415 evolved), they are denoted as Stage 3 samples.

The ^113^Cd NMR signals in Fig. 4b (for Stage 2 CdTe, Stage 2 CdSe, and the mixture of the two) are evidently different from those collected from Stage 1 CdTe, Stage 1 CdSe, and the mixture of the two, together with the Cd precursor Cd(OAc)_2_/OLA (Supplementary Fig. 14). The chemical shifts of the four ^113^Cd NMR signals are similar (in the range of 138–140 ppm). For the three ^113^Cd resonance signals illustrated in Fig. 4b, they are much broader than these four in Supplementary Fig. 14, together with down-field chemical shifts (143–151 ppm). CdTe PCs and CdSe PCs are not formed in Stage 1 CdTe and Stage 1 CdSe, respectively^24,25^. By the way, a recent study documents that the CdSe binary PC of CdSe MSC-415 is white in colour with a composition of 2 Cd to 1Se^43^; we argue that the cause for the enough amount of the PC formed is related to its own formation pathway in the induction period^16,21–26^. The conversion of SeTOP in an induction period (of CdSe QDs with the growth period of less than 5 min) was documented to be around 20%^17^. For the present study, the TeTOP and SeTOP conversion is estimated to be about 37% and 16%, respectively, for Stage 2 CdTe and Stage 2 CdSe (Supplementary Fig. 15).

Thus, the ^113^Cd NMR study (Fig. 4 and Supplementary Fig. 14) is in agreement with the absence and presence of the two binary PCs at relatively low and high temperatures, respectively. Accordingly, the ^113^Cd resonance signal (red trace of Fig. 4b) is indicative of the formation of CdTeSe alloy PCs. Undoubtedly, these Stages 1–3 samples have different amounts of monomers, fragments, and PCs. Stage 1 samples have the Cd precursor and TeTOP or the Cd precursor and SeTOP, without the formation of Cd–Te and Cd–Se covalent bonds^24,25^. Stage 2 samples are induction period samples containing binary PCs and monomers and fragments. Stage 3 samples have undergone the PC → MSC transformation and thus have a smaller number of PCs^16,21–26^.

Figure 5 shows optical absorption spectra collected from nine mixtures made from CdTe and CdSe in equal volumes at room temperature, which provides further support regarding the substitution reactions signified by Equations (2) and (3). Intriguingly, the optimum formation of MSC-399 is found to be the mixture where CdTe Stage 2 and CdSe Stage 2 are mixed, as shown by Fig. 5e (highlighted in red). The strength of the 399 nm peak intensifies, as more MSC-399 is formed due to the PC → MSC transformation (occurring in the mixture up to 4 h incubation at room temperature).

In all other cases, MSC-399 is not observed. There are no absorption peaks seen in Sample a, while for the other samples, MSC-371 and/or MSC-415 characteristic absorption peaks are present. For the mixture (a) of CdTe Stage 1 and CdSe Stage 1, the absence of any MSC characteristic peaks can be attributed to the absence of Cd–Te and Cd–Se bonds, for interactions between the Cd, Te, and Se precursors at room temperature^24,25^. For the mixture (b) of CdTe Stage 1 and CdSe Stage 2, the emergence of a peak at 415 nm indicates the appearance of MSC-415; this is in agreement with that anion exchange reactions of TeTOP and CdSe PCs does not take place during incubation, but the CdSe PC → MSC-415 transformation does. For the mixture (c) of CdTe Stage 1 and CdSe Stage 3, a peak at 415 nm changes little over incubation; thus, TeTOP has no effect on CdSe MSC-415 either.

For the mixture (d) of CdTe Stage 2 and CdSe Stage 1, the gradual appearance of a peak at 371 nm indicates the presence of CdTe MSC-371; this is consistent with the absence of anion exchange reactions of CdTe PCs and SeTOP, and with the presence of the CdTe PC → MSC-371 transformation during incubation. For the mixture (f) of CdTe Stage 2 and CdSe Stage 3, a peak at 371 nm seems to emerge gradually, which indicates that during incubation, the CdTe PC → MSC-371 transition takes place and is not significantly affected by the presence of the CdSe sample. The absence of significant change for the 415 nm peak seems to suggest that MSC-415 is not affected by the CdTe PC → MSC-371 transition.

For the mixture (g) of CdTe Stage 3 and CdSe Stage 1, the 371 nm peak remains essentially constant; thus, MSC-371 appears unaffected by SeTOP. For the mixture (h) of CdTe Stage 3 and CdSe Stage 2, the strength of the 371 nm peak is clearly constant; thus, MSC-371 appears unaffected by CdSe PCs. No 415 nm peak evolves, suggesting that the CdSe PC → MSC-415 transition is slowed down somehow by the presence of the CdTe sample. Finally, for the mixture (i) of CdTe Stage 3 and CdSe Stage 3, the two peaks at 371 and 415 nm are clearly identifiable and constant, indicating that MSC-371 and MSC-415 do not substantially interfere with each other. The formation of the binary MSCs consumes the corresponding PCs; thus, CdTeSe PCs do not form and thus no MSC-399 evolves.

**Fig. 6:**
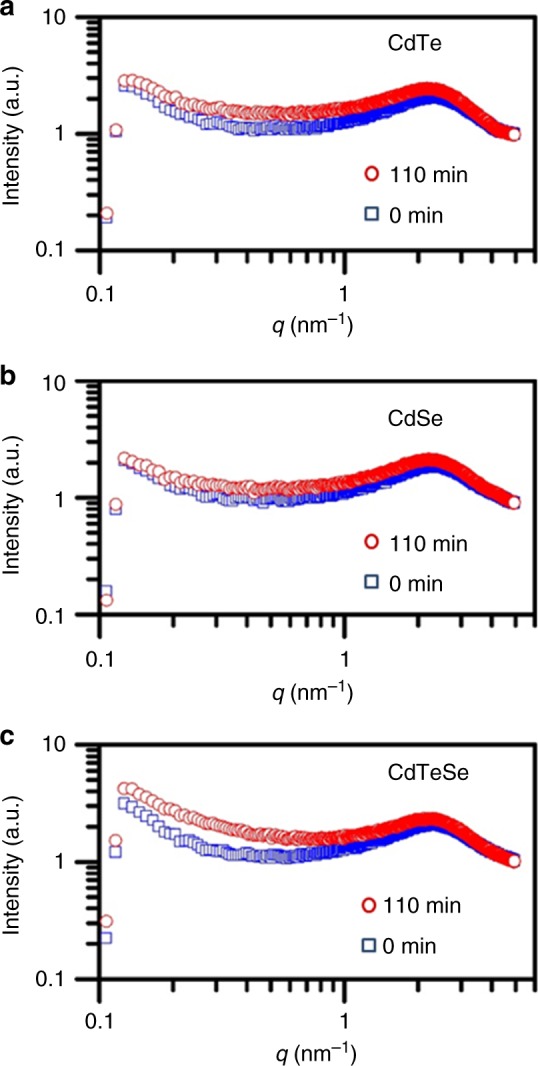
In situ SAXS profiles for two binary samples and their mixture. The binary CdTe (**a**) and CdSe (**b**) samples were reacted at 130 and 140 °C, respectively, for 30 min and the mixture (**c**) was obtained at room temperature by mixing the binary samples with equal volumes. The SAXS data were collected each 10 min up to 110 min, with the spectra of 0 (blue squares) and 110 min (red circles) presented here. The SAXS study illustrates that the size of the three samples are clearly similar (as demonstrated by Supplementary Table 2 and Supplementary Fig. 16)

To further explore the formation pathway of MSC-399, synchrotron-based in situ SAXS is performed. SAXS has been widely used to provide valuable information such as the size of targets of interest^24,44,45^. For the present study, SAXS provides valuable information on the size of induction period samples of binary CdTe and CdSe, together with their room temperature mixtures. The SAXS data are collected each 10 min up to 110 min (Supplementary Fig. 16). With the 0 (blue squares) and 110 min (red circles) spectra presented only, Fig. 6 displays the SAXS profiles collected at room temperature of CdTe (a), CdSe (b), and their mixture (c). The two binary samples are heated for 30 min at 130 °C for CdTe and at 140 °C for CdSe. The peaks at approximately 2 nm^−1^ are attributed to inter-particle scattering from the species in CdTe (a) and CdSe (b) and the mixture (c).

Based on the SAXS profiles obtained from the two binary samples and their mixture (shown in Fig. 6), Supplementary Table 2 summarizes the overall sizes. Up to 110 min, there is a steady increase in the overall size from 0.8 to 1.0 nm for CdTe, 0.8 to 0.9 nm for CdSe, and 0.8 to 1.0 nm for the mixture. Unambiguously, the sizes obtained from the CdTe, CdSe, and mixture samples are quite similar, and the trend with time is similar too. Supplementary Fig. 17 presents the overall size estimated for the binary samples of CdTe, CdSe, and their mixture based on the measurements up to 110 min. Supplementary Fig. 18 presents the corresponding absorption properties of the Fig. 6 samples along with the SAXS data collection. The evolution of MSC-371 and MSC-399 is clearly demonstrated in Parts a and c of Supplementary Fig. 18, respectively.

In general, the SAXS study shows that no larger species formed in the mixture of the CdTe and CdSe samples. Critically, the mixture has a similar size to those of the two binary samples. Therefore, one CdTeSe PC should not be resulted from an addition reaction of one CdTe PC with one CdSe PC, but probably from the substitution reactions symbolized by Equations (2) and (3).

In conclusion, we have effectively synthesized alloy CdTeSe MSC-399 and have explored its formation pathway. Two induction period samples (IPS) of binary CdTe and CdSe are independently prepared at elevated temperatures, and are mixed at room temperature (Equation (1)). The resulting MSC-399 is in a single-ensemble form without the presence of other-bandgap MSCs and QDs. Intriguingly, for a conventional approach consisting of Cd, Te and Se precursors, the reaction is not able to produce the CdTeSe MSCs (Supplementary Fig. 4). To explore the formation pathway, a combination of characterization tools is applied, including optical absorption spectroscopy, ESI-MS, ^113^Cd NMR, and synchrotron-based in situ SAXS. Only one optical absorption peak at 399 nm evolves from the binary mixture after incubation (Fig. 2), suggesting that the CdTe PC → MSC-371 and CdSe PC → MSC-415 transformation do not take place, but the substitution reactions (Equations (2) and (3)) do, together with the CdTeSe PC → MSC-399 transformation (Equation (4)). Our ESI-MS study (Fig. 3) illustrates that the substitution reactions to CdTeSe PC occur quickly; Cd_*x*_Te_*y*_Se_*z*_ fragment species are detected when two induction period samples of binary CdTe and CdSe are mixed at room temperature for 30 min. Furthermore, the formation of Te–Cd–Se covalent bonds at room temperature is supported by ^113^Cd NMR (Fig. 4); the ^113^Cd resonance signal of a mixture of two induction period samples of binary CdTe and CdSe is located between those of the two binary samples. Moreover, the size of a binary mixture obtained is similar to those of corresponding binary CdTe and CdSe, as suggested by SAXS (Fig. 6). Therefore, it seems reasonable to hypothesize that the formation of CdTeSe MSC-399 at room temperature does not come from the addition of CdTe MSC-371 and CdSe MSC-415, but is via its own precursor compound CdTeSe PC-399 (Equation (4)). The formation of CdTeSe PC-399 at room temperature is neither via partial anion exchange reactions (of CdTe PC + SeTOP or CdSe PC + TeTOP, Fig. 5 and Supplementary Fig. 13), nor via the addition reaction of CdTe PC-371 and CdSe PC-415, but is via the substitution reactions (Equations (2) and (3)). Exploring the chemistry at the nanoscale, the present effort on CdTeSe MSC-399 narrows the knowledge gap on the synthesis and formation pathway of ternary MSCs at room temperature. It is probable that the room temperature approach to ternary CdTeSe MSC-399 developed and the formation pathway proposed in present study is applicable to other ternary semiconductors MSCs such as CdTeS. We are actively exploring the applicability of the approach, together with the structure of binary MSCs using Random Structure Searching methods with DFT calculations^46^. The presence of a protic agent such as methanol or a primary amine has been documented to accelerate the binary PC to binary MSC transformation^16,21–26,47–49^. For the formation of CdSe MSC-415 (from CdSe QDs) via the presence of a primary amine^49^, the present study enables us to comprehend the pathway to be probably via CdSe PC-415 from the QD fragmentation; however, in-depth physical insight for the process still requires more efforts (such as the exploration of activation energy). This subject will be our forthcoming study, which will be also extended to alloy MSCs. Given the complexity of the subject, it is impossible to extract complete mechanistic insight from one study. Even so, the present study is one solid piece (in a very large puzzle), providing the formation pathway of the alloy MSCs and making the synthesis less empirical. We believe that the field of colloidal nanocrystals is transforming, as it must, from an empirical art to science, similar to the advance of organic chemistry^50–55^.

## Methods

### Chemicals

Cadmium acetate dihydrate (Cd(OAc)_2_•2H_2_O 99.999%, Alfa Aesar), tellurium powder (99.99%, Alfa Aesar), selenium powder (99.99%, Alfa Aesar), oleylamine (OLA, 90%, Aldrich), tri-*n*-octylphosphine (TOP, 90%, Aldrich), octylamine (OTA, 99%, Aldrich), ethylacetate (99.5%, Tianjin Zhiyuan Chemical), and nitrogen gas (N_2_, 99.99%, Chengdu Taiyu gas Co. Ltd.) were used as received without further purification unless stated otherwise. Toluene (AR grade, Chengdu Kelong Chemical) was distilled and further dried with MgSO_4_ (99%, Tianjin Zhiyuan Chemical). Oleylamine (OLA) was stored in a freezer, while the other chemicals were stored under an ambient environment.

### Cd precursor preparation

Cd(OAc)_2_•2H_2_O (0.160 g, 0.6 mmol) and OLA (3.000 g) were added into a 50 mL three-necked flask at room temperature, and the flask was evacuated and then purged with N_2_ gas; this procedure was repeated three times until no bubbles were observed under vacuum. Then, the mixture was heated up to 80 °C under a N_2_ atmosphere, followed by evacuation for an hour until no bubbles were apparent. Under a N_2_ atmosphere, the mixture was heated up to 120 °C, and was kept under vacuum for 1 h. In this way, our Cd precursor was obtained in the form of a clear, light yellow solution.

### Preparation of Te and Se precursors

The Te and Se precursors were tri-*n*-octylphosphine telluride (TeTOP) and tri-*n*-octylphosphine selenide (SeTOP), respectively, with a feed molar ratio of 4TOP to 1E (E = Te or Se). Tellurium powder (0.019 g, 0.15 mmol) or Selenium powder (0.012 g, 0.15 mmol) and TOP (0.247 g, 0.6 mmol) was added into a 25 mL three-necked reaction flask at room temperature. The mixture was degassed under vacuum and purged with N_2_ gas; this process was repeated three times. Under a N_2_ environment, the Te mixture was heated to 300 °C for 30 min, while the Se mixture was heated to 40 °C for 10 min. When the two clear solutions were cooled to room temperature, OLA (1.574 g for TeTOP and 1.581 g for SeTOP) was added (in order to reach a total weight of 5.00 g for the binary sample preparation).

### Preparation of CdTe and CdSe samples and their mixtures

Reactions were carried out with a 4 Cd to 1E (E = Te or Se) feed molar ratio, and a Cd concentration of 120 mmol·kg^−1^ (96 mM) in OLA. The Cd precursor solution at 120 °C was mixed with the E precursor solution, resulting in a total weight of about 5.00 g. The resulting two mixtures were degassed under vacuum until no bubbles were apparent; under a N_2_ atmosphere, the mixtures were heated for 30 min at a desired temperature (120–140 °C). Clear solutions were obtained. Occasionally, the samples were stored at a liquid N_2_ temperature for future use. The CdTe and CdSe samples were mixed at room temperature with a 1:1 volume ratio in a vial. For the formation of CdTeSe MSC-399, incubation was required.

### Optical absorption measurements

An aliquot of each sample (30 μL) was dispersed in toluene (3.0 mL). The measurements were performed on a Hitachi UH4150 spectrometer and the spectra were usually collected in the range of 300–600 nm with a 1 nm interval. Toluene was measured as a background sample. Quartz cuvettes from Hellma Analytics (with the light path of 10 mm of 3.5 mL standard QS cells) were used.

### Electrospray ionization mass spectrometry (ESI-MS)

An Agilent 6210 A HPLC-TOF/MS in a positive ion mode was used, with acetonitrile as a mobile phase. For the operation of the instrument and the followed-up data analyses, Agilent Mass Hunter software was used. By a side note, the binary samples were placed in a liquid N_2_ temperate for 3 days. For their mixture, it was prepared at room temperature and incubated for about 30 min. The resulting mixture sample was then stored in a liquid N_2_ temperate for 3 days. Before measurements, the frozen binary samples and their mixture were de-frozen at room temperature, and 30 μL of each was dispersed in toluene (3.0 ml).

### ^113^Cd nuclear magnetic resonance (NMR)

As-synthesized CdTe and CdSe samples (0.3 mL), together with their mixture (0.3 mL), were diluted with 0.3 mL toluene-*d*_*8*_ in a glovebox. The mixture was prepared with the same volumes of the two binary samples at room temperature, and was incubated for 15 min. Each of the spectra was collected with a Bruker Avance III 400 MHz, with Cd(ClO_4_)_2_ as a chemical shift reference and a scanning number of 4096^24^. It took about 4 h to collect each of the spectra. See Supplementary Table 1 for details.

### Synchrotron-based in situ small angle X-ray scattering (SAXS)

The binary CdTe (130 °C/30 min) and CdSe (140 °C/30 min) samples were used (with the Cd concentration of 96 mM). They were then stored in a liquid N_2_ temperature overnight. The next day, the binary samples were mixed at room temperature with equal volumes. The SAXS measurements were carried out at room temperature after the samples added into sample holders with a light pathlength of about 1.0 mm; no dilution was performed. Again^24^, the BL16B1 beamline at Shanghai Synchrotron Radiation Facility (SSRF), Shanghai, China, was used, with X-rays of a wavelength of λ = 1.03 Å (energy of 12 keV) as the incident beam. A Rayonix SX-165 CCD detector (Rayonix, Evanston, IL, USA) with a resolution of 2048 × 2048 pixels and a pixel size of 80 μm × 80 μm was used to record the scattering intensity. Importantly, all of the data were corrected for background and air scattering. Similarly^24^, the two-dimensional pattern was integrated to obtain the one-dimensional SAXS profile with Fit2D software. The detailed information about SAXS data fitting with a model developed by Beaucage can be found elsewhere^24^.

## Supplementary information

Supplementary Information

## Data Availability

The authors declare that all relevant data supporting the findings of this study are available from the authors on request.
