# Supplementary material for: Formation of colloidal alloy semiconductor CdTeSe magic-size clusters at room temperature
Source: Nat Commun. 2019 Apr 11;10:1674. doi: 10.1038/s41467-019-09705-w (PMC6459852; doi:10.1038/s41467-019-09705-w)
Supplement: Supplementary file 1 — Supplementary Information [file 41467_2019_9705_MOESM1_ESM.pdf]

Formation of Colloidal Alloy Semiconductor CdTeSe

Magic-Size Clusters at Room Temperature

Yu* et al.

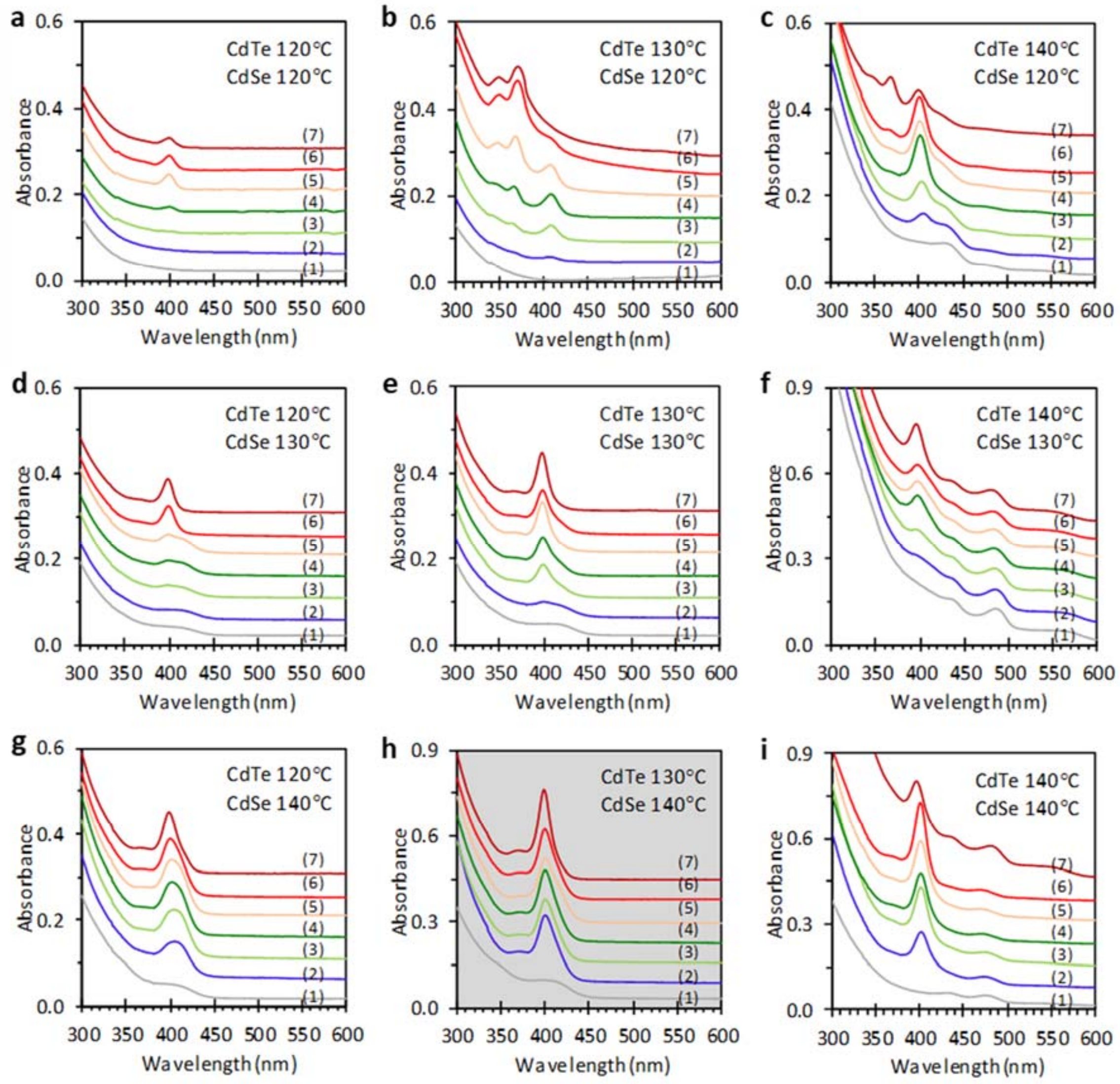

Supplementary Figure 1. Optical absorption properties for the nine mixtures made from the binary samples of CdTe and CdSe, which were heated for 30 min at the temperatures indicated. The binary samples were mixed with equal volumes at room temperature. The resulting mixtures were incubated for 0 min (1, grey traces), 30 min (2, blue traces), 1 h (3, light green traces), 2 h (4, dark green traces), 4 h (5, orange traces), 8 h (6, red traces), and 20 h (7, burgundy traces). An aliquot (30 µL) of each of the nine mixtures was dispersed in toluene (3.0 mL). Absorption measurements were then performed. Clearly, the binary

samples in the left and middle columns (a, b, d, e, g and h) are induction period samples, while nucleation and growth took place in the CdTe 140 °C samples in the right column, as suggested by the presence of quantum dots (QDs). It seems that one optimum preparation of MSC-399 is from the CdTe 130 °C and CdSe 140 °C samples (h, as highlighted in a grey color). Careful observation suggests that the 4-h incubation (orange traces) seems to have the evolution of CdSe MSC-415 (such as the d, g, and h). The disappearance of CdSe MSC-415 for the corresponding longer incubation samples is in agreement with the reaction of CdSe MSC-415 → CdSe PC[1] and the substitution reaction described by CdSe PC + CdTe M/F → CdTeSe PC.

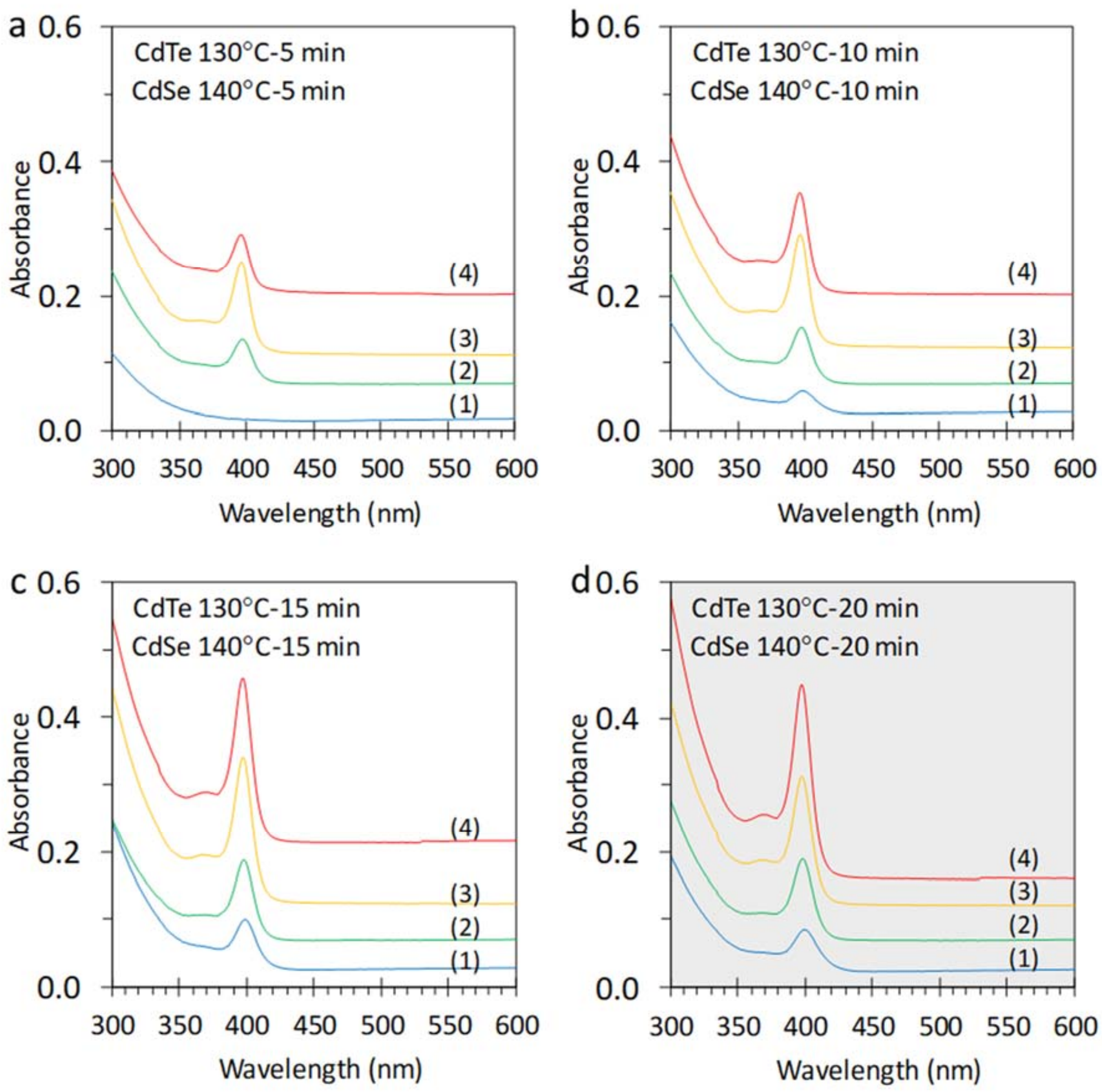

Supplementary Figure 2. Optical absorption spectra collected from the four mixtures made from the two binary induction period samples of CdTe (heated at 130 °C) and CdSe (heated at 140 °C). The reaction periods are indicated. The binary samples were mixed with equal volumes at room temperature. The resulting mixtures were incubated for (1) 1, (2) 2, (3) 3, and (4) 4 h. An aliquot (30 µL) of each of the nine mixtures was dispersed in toluene (3.0 mL). Absorption measurements were then performed. Evidently, the 20 min heated period (d, as highlighted in a grey color) seems to be better than the shorter periods explored, with regard to the amount of MSC-399 obtained. The effect explored is in agreement with the substitution reactions proposed. The more CdTe and CdSe PCs are produced, the more MSC-399 is obtained.

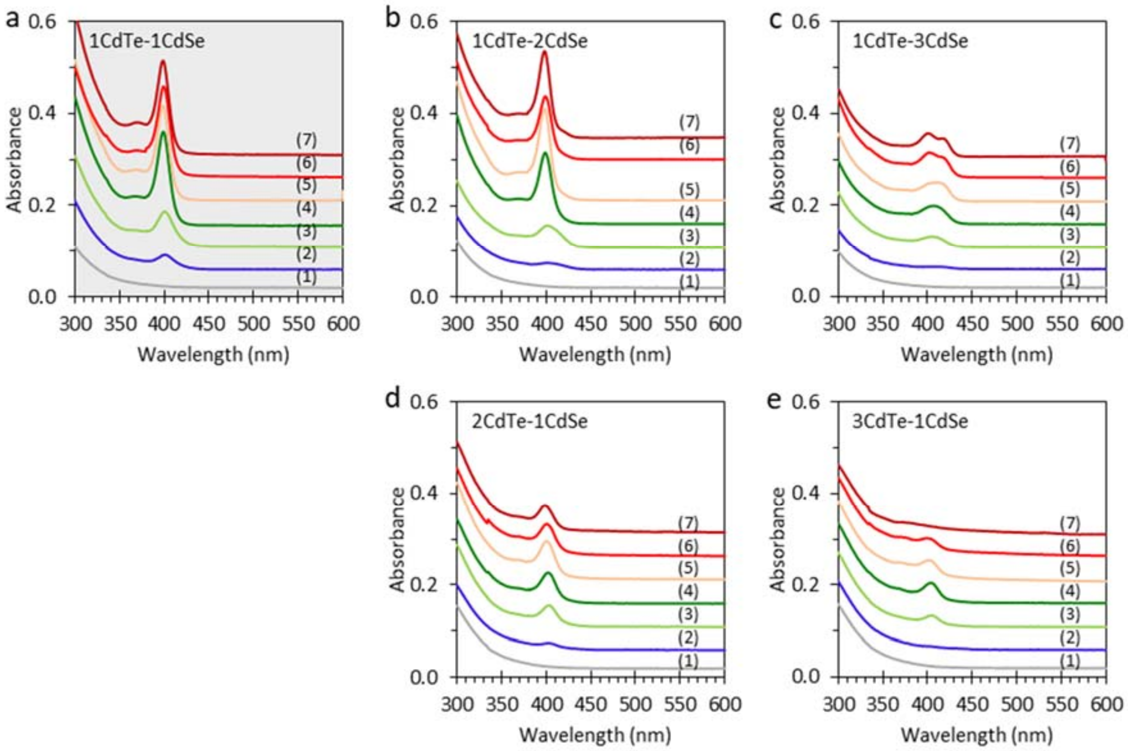

Supplementary Figure 3. Optical absorption spectra collected from the five mixtures made from the two binary induction period samples of CdTe 130 °C/30 min and CdSe 140 °C/30 min. The binary samples were mixed at room temperature with the volume ratios indicated. The resulting mixtures (with the total volume of 1.0 mL) were incubated for (1) 0 min, (2) 30 min (3) 1 h, (4) 7 h, (5) 22 h, (6) 30 h and (7) 48 h. An aliquot (30 μL) of each of the nine mixtures was dispersed in toluene (3.0 mL). Absorption measurements were then performed. Apparently, the 1 to 1 volume ratio (a, as highlighted in a grey color) seems to be better, for the amount of MSC-399 evolved. This is in agreement with the larger amount of CdTe and CdSe PCs produced. The volume ratio effect is in agreement with the substitution reactions of CdTe and CdSe PCs proposed.

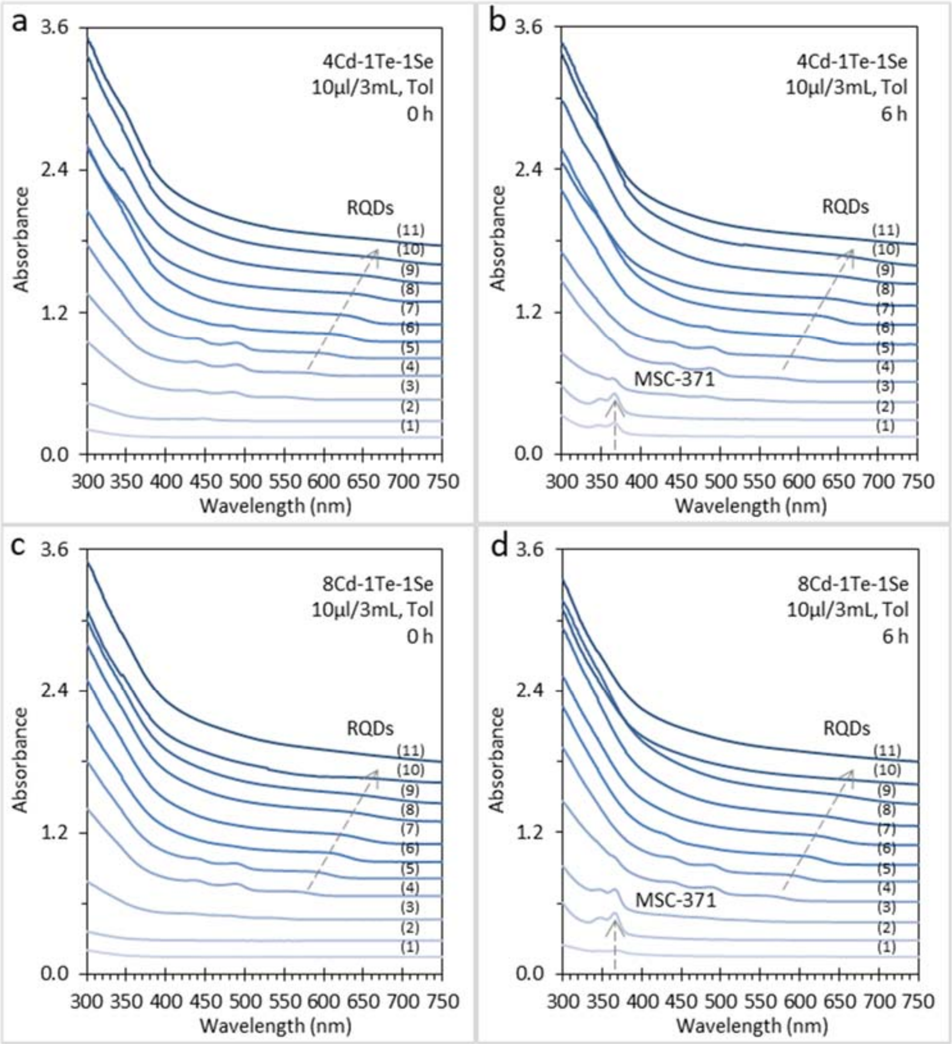

Supplementary Figure 4. Evolution of absorption properties of the samples from heating up reaction batches of 4Cd(OAc)$_2$/OLA + 1TeTOP + 1SeTOP (a and b) and 8Cd(OAc)$_2$/OLA + 1TeTOP + 1SeTOP (c and d), with the Cd concentrations at 120 and 240 mmol kg^{-1}, respectively. Eleven samples were extracted at (1) 120, (2) 130, (3) 140, (4) 150, (5) 160, (6) 170, (7) 180, (8) 190, (9) 200, (10) 210 and (11) 220 °C with the 15-min reaction period at each temperature. These samples were then incubated at room temperature for 0 h (a and c) and 6 h (b and d), as indicated. An aliquot (10 µL) of each of these samples was dispersed in toluene (Tol, 3.0 mL), and its spectrum was collected. Intriguingly, CdTe MSC-371 was obtained in the incubated samples but no MSC-399.

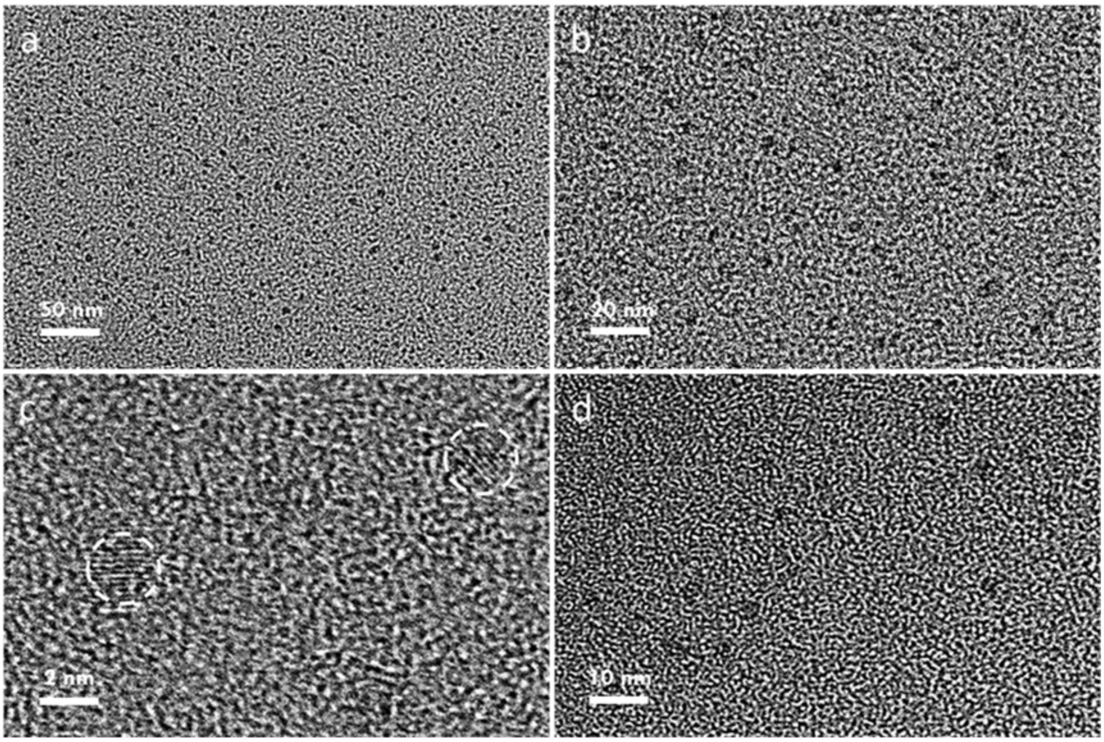

Supplementary Figure 5. Transmission electron microscope (TEM) images of our as-synthesized alloy CdTeSe MSCs. For the study, the binary induction period sample of CdTe (130 °C/30min) and CdSe (140 °C/30min) were mixed at room temperature with equal volumes. The mixture was then incubated for two days to allow the formation of CdTeSe MSC-399. An aliquot (100 µL) of the mixture sample was dispersed in toluene (3.0 mL) for about 1 min; the resulting dispersion was drop cast on a TEM grid. The TEM grid was placed in a fume-hood for 30 min to accelerate the evaporation of solvent. The images were taken on JEM-2100F at Southwest Jiaotong Univ (a and b) and at Fudan Univ (c and d).

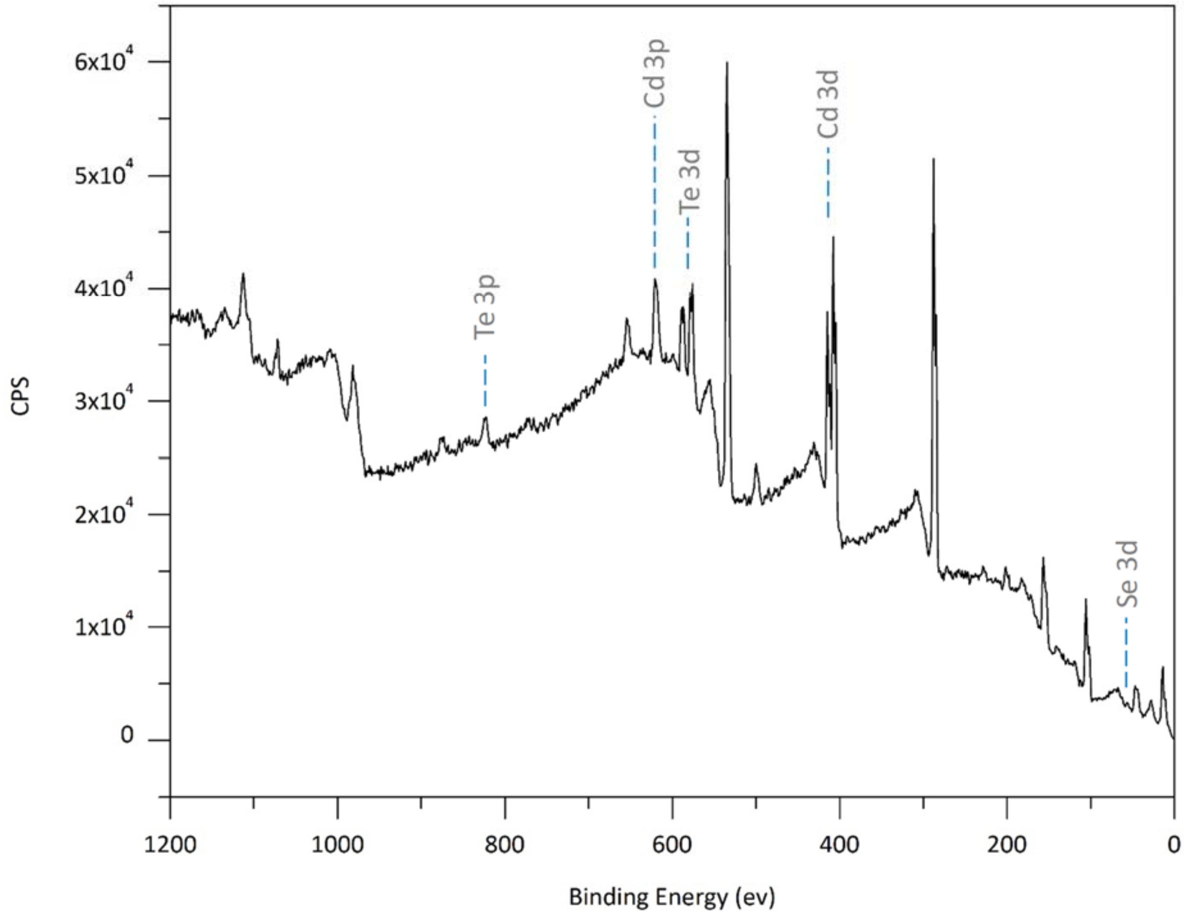

Supplementary Figure 6. An example of XPS study of alloy CdTeSe MSC-399 purified. The purification was performed three times with toluene and ethylacetate. Centrifugation at room temperature with a speed of 9000 rpm for 15 min was carried out on a Beckman Coulter Allegra 64R Centrifuge. The final precipitate was dispersed in toluene for the preparation of the XPS sample. XPS study illustrates the presence of Cd, Te, and Se.

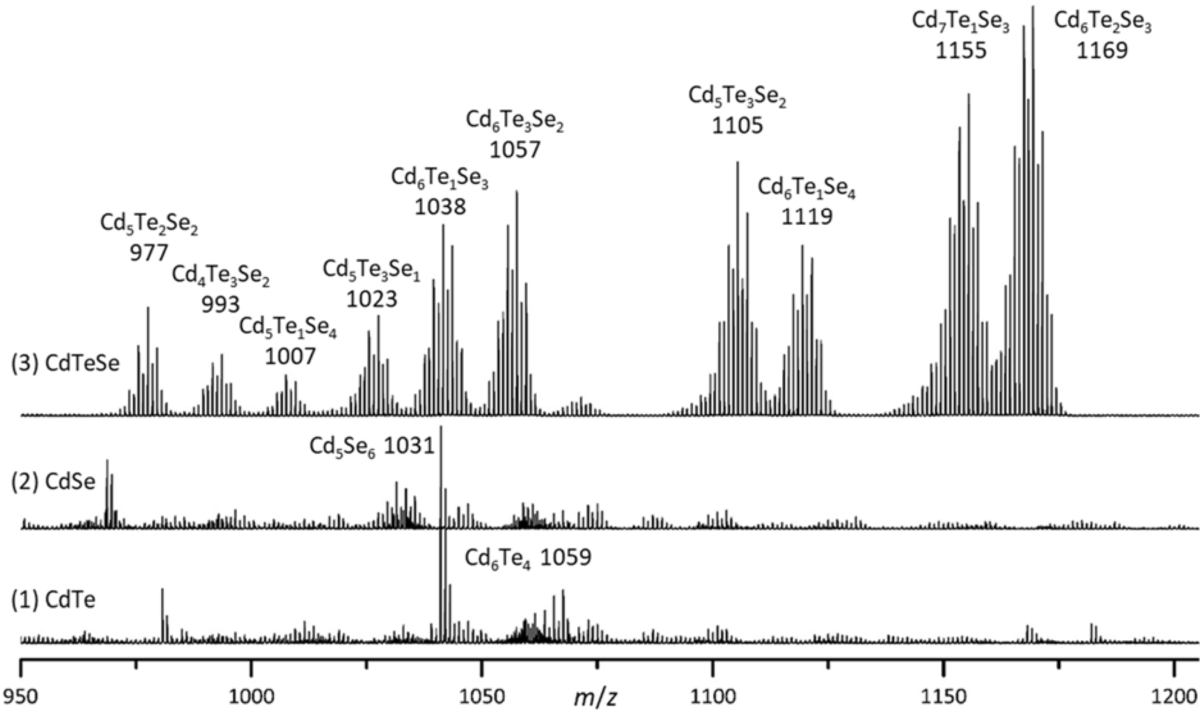

Supplementary Figure 7. ESI-MS spectra of the Fig. 2 binary samples of CdTe (trace 1) and CdSe (trace 2), together with the Fig. 2 mixture (trace 3), in the *m/z* region of 950-1250 Da. The fragments detected in traces (1) and (2) are attributed to the presence of CdTe and CdSe PCs, respectively. The various $Cd_xTe_ySe_z$ fragments monitored in trace (3) suggest the presence of Te-Cd-Se bonds.

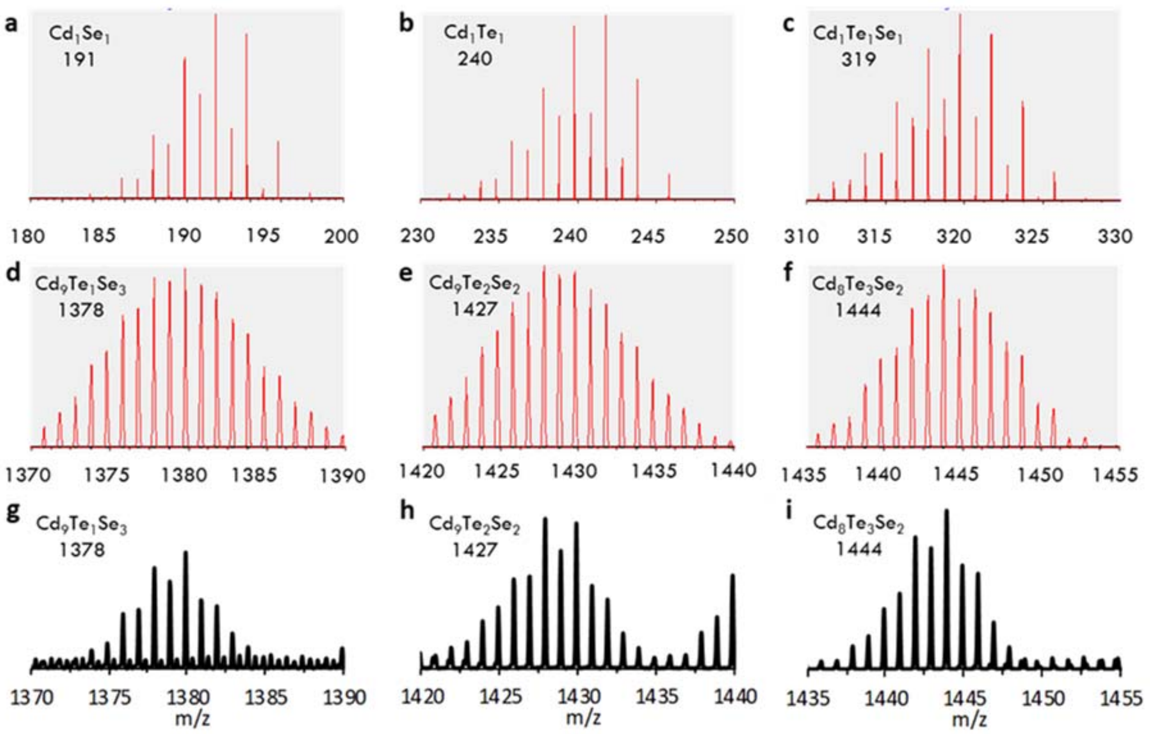

Supplementary Figure 8. The isotope distribution of $Cd_{1}Te_{1}$ (a) and $Cd_{1}Se_{1}$ (b) obtained from Isopro software with Lorentz fitting, together with that of $Cd_{1}Te_{1}Se_{1}$ (c). $Cd_{9}Te_{1}Se_{3}$ (d), $Cd_{9}Te_{2}Se_{2}$ (e), and $Cd_{8}Te_{3}Se_{2}$ (f) simulated. $Cd_{9}Te_{1}Se_{3}$ (g), $Cd_{9}Te_{2}Se_{2}$ (h), and $Cd_{8}Te_{3}Se_{2}$ (i) detected by MS; as shown by Fig. 2, they have larger peak heights. The simulation and experimental data match well with each other; thus, our assignment should be quite rational. The consistency between the MS and other characterization tools adds credibility to our proposed substitution mechanism for the formation of the precursor compound (PC-399) of alloy MSC-399.

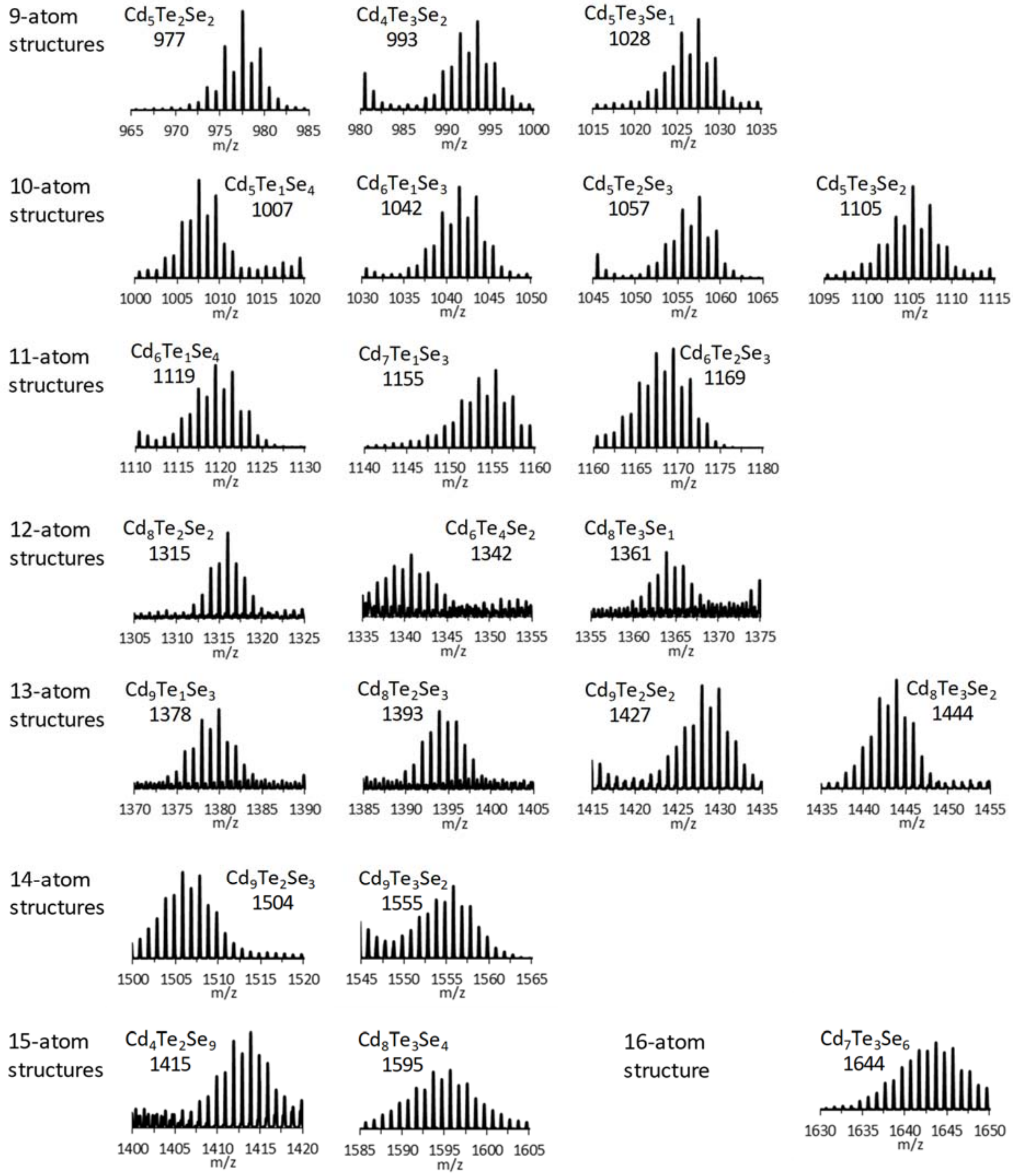

Supplementary Figure 9. The summary of the fragments of alloy CdTeSe PCs detected from the *m/z* region of 900–1700 Da.

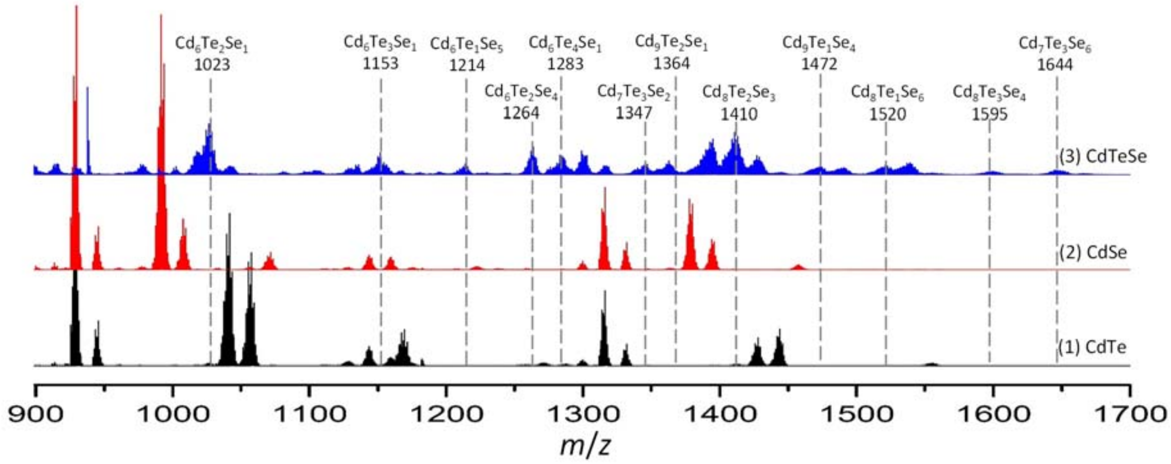

Supplementary Figure 10. ESI-MS spectra of the binary samples of CdTe 130 °C/30 min (trace 1) and CdSe 140 °C/30 min (trace 2) (without incubation), together with their room-temperature and equal-volume mixture (with 30 min incubation) (trace 3), in the *m/z* region of 900-1700 Da. There are fragments detected in trace (3), which are not shown in traces (1) and (2), suggesting the formation of Te-Cd-Se covalent bonds.

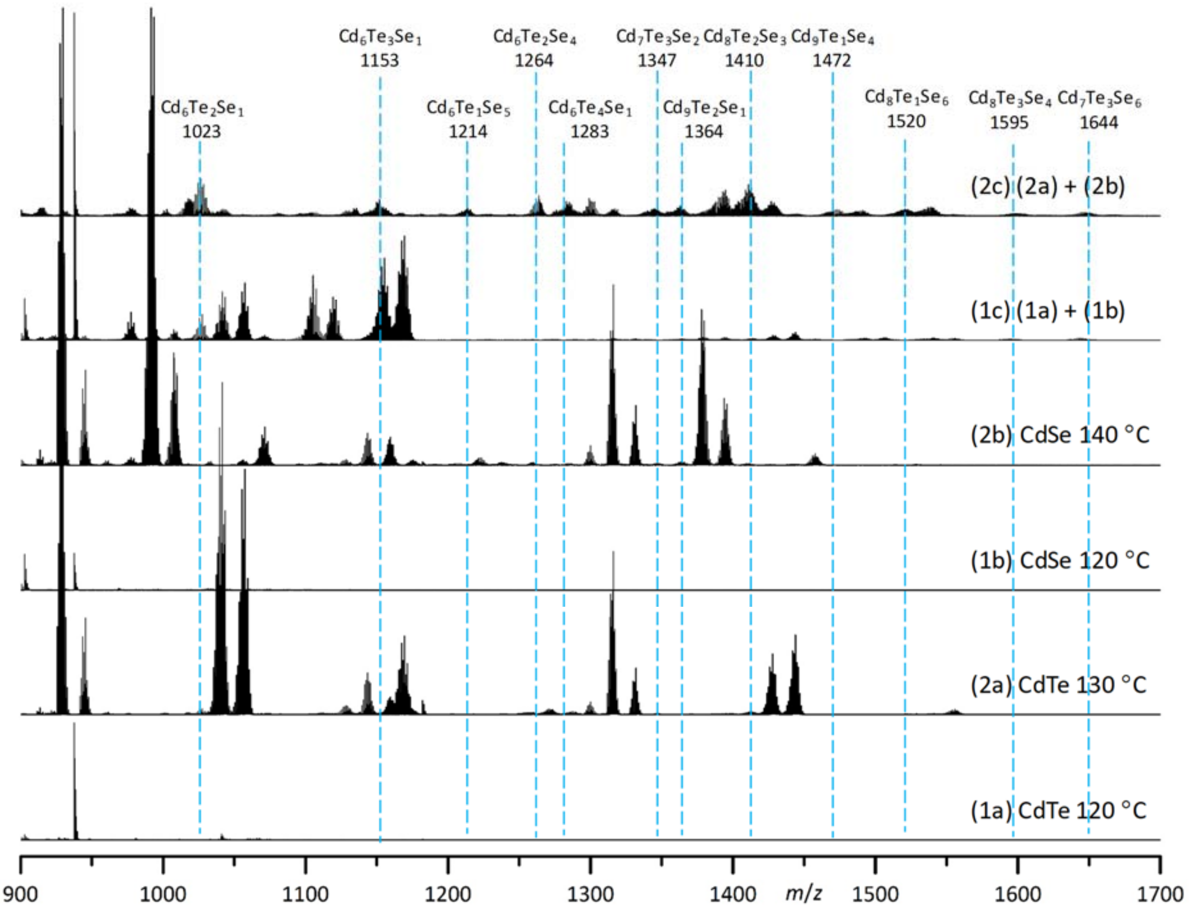

Supplementary Figure 11. ESI-MS spectra of CdTe (a), CdSe (b) induction period samples and their mixtures with equal volumes (c) with a positive-ion mode in the *m/z* region of 900-1700. The CdTe and CdSe samples were obtained from reaction batches with a reaction period of 30 min at 120 °C (1a, 1b), or at 130 °C (2a) and 140 °C (2b). Traces (a) and (b) show the signal of Cd_xTe_y and Cd_xSe_y fragments, respectively. Trace (c) shows a class of fragments which were not observed in traces (1) and (2), suggesting the formation of Te-Cd-Se covalent bonds upon mixing CdTe and CdSe induction period samples.

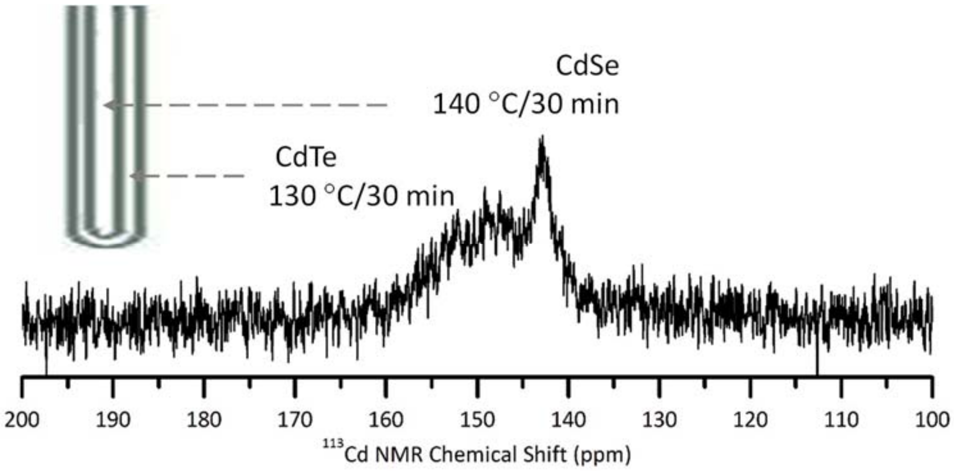

Supplementary Figure 12. ^{113}Cd NMR spectrum collected from the CdTe 130 °C/30 min and CdSe 140 °C/30 min samples. The two samples were diluted with the same volume toluene-d_8. The diluted CdSe sample (300 µL) was added into an NMR tube with an outer diameter of 2.5 mm, which was inserted into another NMR tube with an outer diameter of 5 mm filled with the diluted CdTe sample (300 µL). Evidently, the spectrum exhibits two peaks at ~151.8 and ~143.0 ppm. The ^{113}Cd resonance signals correspond to the CdTe and CdSe samples, respectively, and are similar to those shown in of Fig. 3b. Accordingly, the two peaks collected from the two mixtures shown in Fig. 3 can be attributed to the formation of Te-Cd-Se covalent bonds, via the substitution reactions proposed.

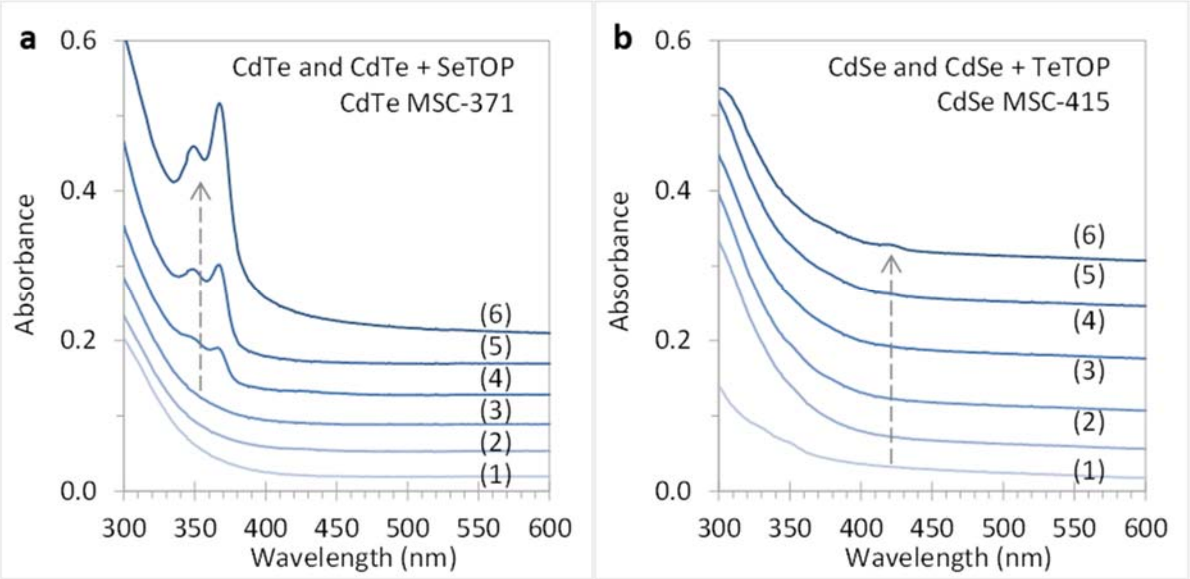

Supplementary Figure 13. Absorption spectra collected, (a) from one CdTe 130 °C/30 min sample (trace 1) and from the mixture CdTe + SeTOP (traces 2 to 6), and (b) from one CdSe 130 °C/30 min sample (trace 1) and from the mixture CdSe + TeTOP (traces 2 to 6). The mixing periods were 0 min (traces 1 and 2), 30 min (traces 3), 1 h (traces 4), 2 h (traces 5), and 4 h (traces 6). An aliquot (30 µL) of each sample was dispersed in toluene (3.0 mL). The presence of CdTe MSC-371 (a) and possible CdSe MSC-415 (b), together with the absence of CdTeSe MSC-399 in the two 4-h mixtures, suggests that anion exchange reactions (with SeTOP and TeTOP) did not take place at room temperature.

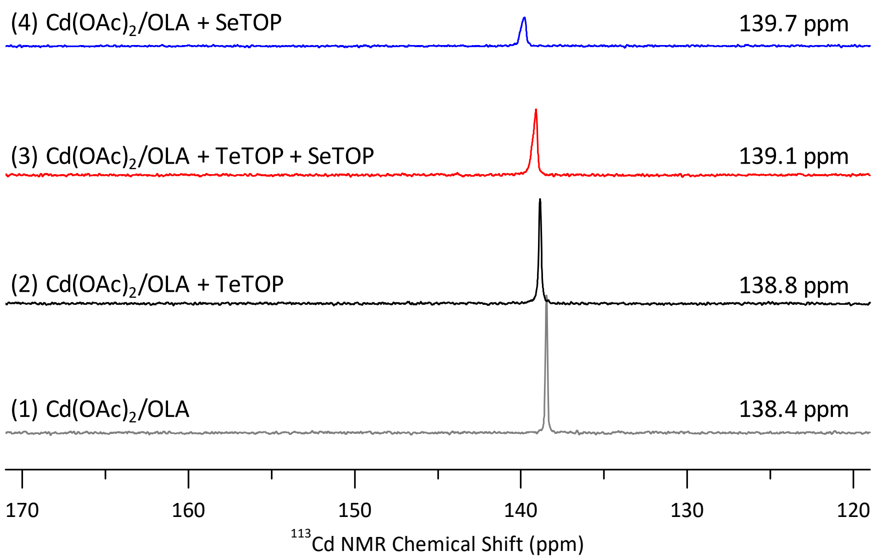

Supplementary Figure 14. The ^{113}Cd NMR spectra collected at room temperature from (1, light grey trace) our Cd precursor Cd(OAc)$_2$/OLA, (2) Stage 1 CdTe (black trace, with the feed molar ratio of 4Cd(OAc)$_2$/OLA to 1TeTOP), (3) a mixture of Stage 1 CdTe and Stage 1 CdSe (red trace, of 8Cd(OAc)$_2$/OLA and 1TeTOP and 1SeTOP), and (4) Stage 1 CdSe (blue trace, with the feed molar ratio of 4Cd(OAc)$_2$/OLA and 1SeTOP). The last three samples were prepared at room temperature in an identical manner to those presented in Fig. 3, but without heating. Traces 2 and 4 suggest that there are almost no CdTe PCs and CdSe PCs formed in the two Stage 1 binary samples. Such absence of the PC is consistent with the fact that there were no fragments detected in the ESI-MS spectra of Stage 1 CdTe and of a mixture of the Cd + Se precursors (heated at even 80 °C from room temperature)[2,3]. Trace 3 suggests there are almost no CdTeSe PCs formed at room temperature from the mixture of the two Stage 1 binary samples; furthermore, the red trace in Fig. 3b demonstrates that CdTeSe PCs form in the mixture of the two Stage 2 binary samples.

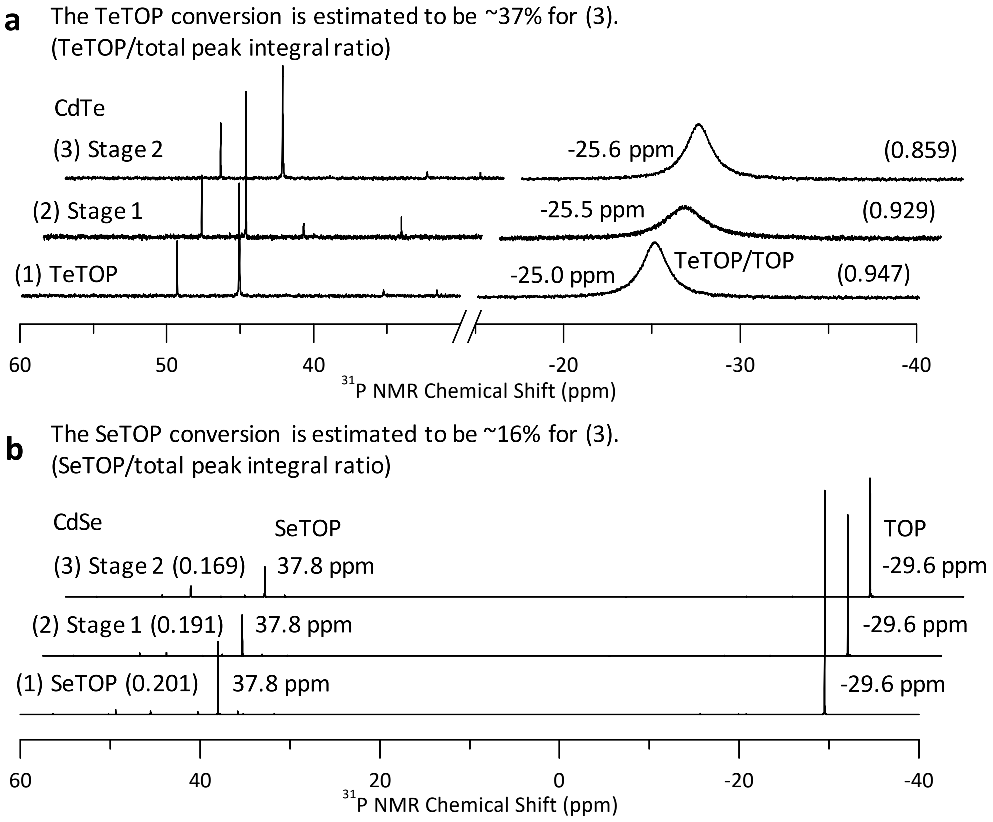

Supplementary Figure 15. Estimation of the conversion of TeTOP (a) and SeTOP (b) for our induction period samples (IPS) also called Stage 2 samples. We used ^{31}P NMR for the conversion estimation[4]. The preparation of the Cd precursor, TeTOP, and SeTOP is identical to that used for Figure 1; also, the synthetic conditions for the four binary Stages 1 and 2 samples are identical to those used for Figure 1. The three samples in the top part (a) and those in the bottom part (b) have the same TeTOP and SeTOP concentrations in toluene-d_8, respectively. For the three spectra in the top part (a), the peaks around -25.6 and -25.0 ppm correspond to TeTOP/TOP[2,5]. For the three spectra in the bottom part (b), the peaks around 37.8 and -29.6 ppm are attributed to SeTOP and TOP, respectively[5-8]. From the ratio of the integral of the TeTOP/TOP peak or SeTOP to that of the total P-related peaks, the TeTOP or SeTOP conversion is estimated to be ~37% or ~16%, respectively. By a side note, the SeTOP conversion in an induction period of CdSe QDs was documented to be around 20% (= (0.060 - 0.048)/0.060 when the reaction was less than 5 min)[4]. Also, the conversion of CdS MSC-360 was claimed to be ~90%[9], while an approach with high precursor concentrations to CdS MSC-322 was reported[10]. These two reports on CdS MSCs suggest that a majority of Cd and

S precursors are able to self-assemble (in the pre-nucleation stage) to result in MSCs[1,11]; accordingly, it is reasonable that the conversion yield (regarding the Cd-E covalent bond formation) in the pre-nucleation stage of a reaction can be high.

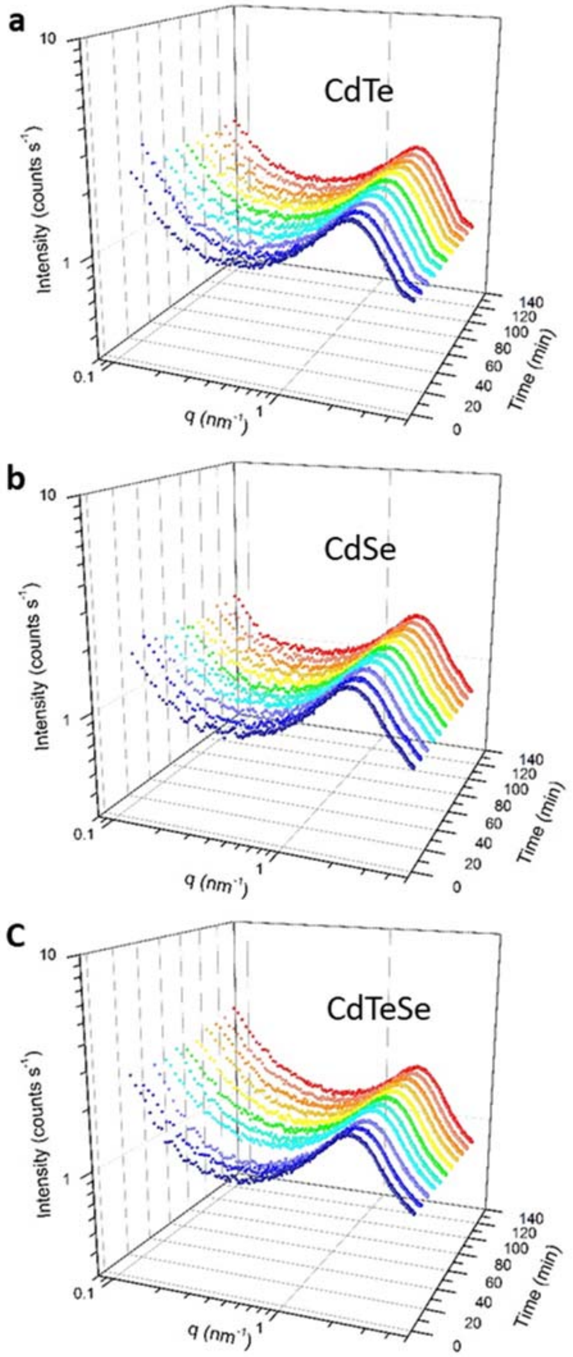

Supplementary Figure 16. *In-situ* SAXS data collected between 0 to 110 min for the binary induction period samples of CdTe 130 °C/30 min (a) and CdSe 140 °C/30 min (b), and their mixture (c).

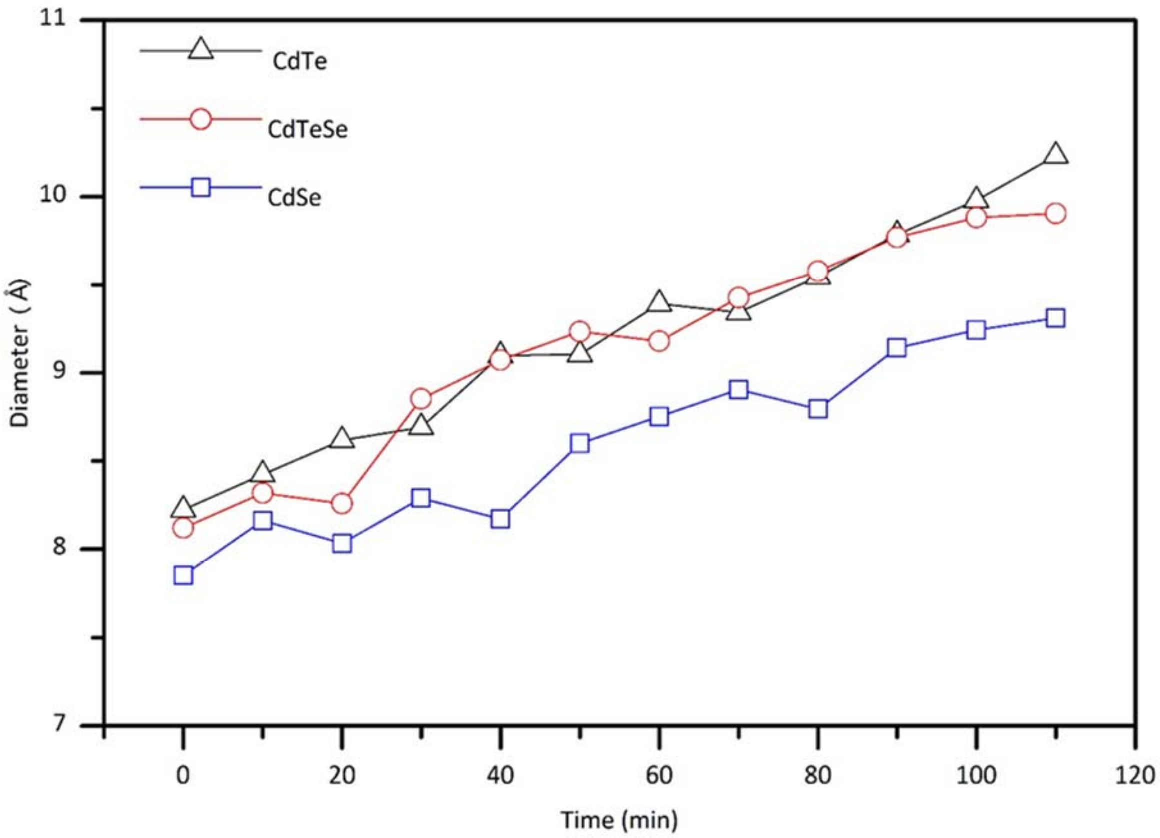

Supplementary Figure 17. Temporal evolution of the overall sizes estimated for the two binary samples of CdTe 130 °C/30 min (triangular black symbols) and CdSe 140 °C/30 min (square blue symbols), together with their mixtures (circular red symbols). The sizes of the three samples are similar. Interestingly, the CdTeSe diameter seems to be closer to that of CdTe. By a side note, the diameter ratio of CdTe to CdSe is similar to the lattice constant ratio of zinc blende CdTe (6.5 Å) to CdSe (6.1 Å)[12,13].

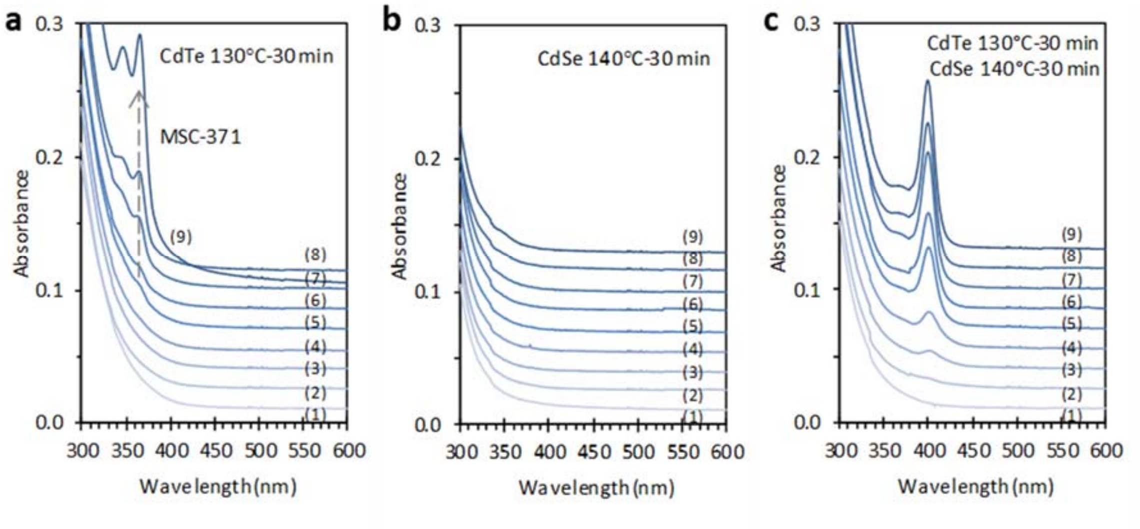

Supplementary Figure 18. Absorption spectra of the Fig. 5 CdTe (a) and CdSe (b) and the mixture (c). The 3 samples were incubated for (1) 0, (2) 10, (3) 20, (4) 30, (5) 40, (6) 50, (7) 60, (8) 80 and (9) 120 min and then measured. An aliquot (30 µL) of each sample (along incubation up to 120 min) was extracted and dispersed in toluene (3.0 mL) at room temperature for the measurement.

Supplementary Table 1. Parameters of ^{113}Cd NMR measurements

Parameters	^{113}Cd NMR
Solvent	Toluene-d_8
Temperature	301.2 K
Pulse Sequence	zgig
Number of Scans	4096
Receiver Gain	35.85
Relaxation Delay	3.0000 sec
Pulse Width	11.5080
Spectrometer Frequency	88.8015510 MHz
Standards	$Cd(ClO_4)_2$

Supplementary Table 2. Estimated diameters for the binary samples of CdTe and CdSe, together with their mixture, based on their SAXS profiles. The SAXS data were collected each 10 min up to 110 min.

	Diameter/Å					
Duration	CdTe	Fitting Error	CdSe	Fitting Error	CdTeSe	Fitting Error
0 min	8.23	0.04	7.86	0.03	8.13	0.03
10 min	8.43	0.03	8.17	0.03	8.32	0.03
20 min	8.62	0.04	8.04	0.05	8.26	0.03
30 min	8.70	0.04	8.30	0.03	8.86	0.03
40 min	9.10	0.02	8.18	0.05	9.08	0.03
50 min	9.11	0.03	8.60	0.03	9.24	0.05
60 min	9.40	0.02	8.76	0.02	9.18	0.03
70 min	9.35	0.03	8.91	0.04	9.43	0.04
80 min	9.55	0.02	8.80	0.04	9.58	0.03
90 min	9.79	0.03	9.15	0.03	9.77	0.03
100 min	9.99	0.02	9.25	0.02	9.88	0.04
110 min	10.24	0.03	9.32	0.03	9.91	0.03

Supplementary Note 1. Explanation of our assignments for those MS clusters detected.

ESI has only relatively recently been employed to the study of induction period samples of colloidal semiconductor ME QDs[2,3,11]. The induction period samples, in particular, do not contain QDs but are composed of other species, including monomers, fragments, and MSC precursor compounds (PCs). The species detected by ESI have masses that correspond to the calculated weights for Cd_xTe_2 (x = integer 5 to 7)[2], Cd_xSe_2 (x = integer 6 to 8)[3], and Zn_xSe_8 (x = integer 4 to 11)[11]. As the variation in the measured masses appears to consist of the change of one Cd or one Zn atomic mass, it seems reasonable that the species detected are free of surface ligands. Furthermore, we would have expected that, for the oxidation state, M is +2 and E is -2; thus, it is curious that the oxidation state of the detected bare species is +1. The oxidation state of the detected species deserves future investigation.

Supplementary References

1. Zhang, J. et al. Individual pathways in the formation of magic-size clusters and conventional quantum dots. *J. Phys. Chem. Lett.* **9**, 3660–3666 (2018).
2. Liu, M. et al. Probing intermediates of the induction period prior to nucleation and growth of semiconductor quantum dots. *Nat. Commun.* **8**, 15467 (2017).
3. Zhu, D. et al. Interpreting the ultraviolet absorption in the spectrum of 415 nm-bandgap CdSe magic-size clusters. *J. Phys. Chem. Lett.* **9**, 2818–2824 (2018).
4. Owen, J. S., Chan, E. M., Liu, H. & Alivisatos, A. P. Precursor conversion kinetics and the nucleation of cadmium selenide nanocrystals. *J. Am. Chem. Soc.* **132**, 18206–18213 (2010).
5. Yu, K., et al. General low-temperature reaction pathway from precursors to monomers before nucleation of compound semiconductor nanocrystals. *Nat. Commun.* **7**, 12223 (2016).
6. Yu, K., et al. Effect of tertiary and secondary phosphines on low-temperature formation of quantum dots. *Angew. Chem. Int. Ed.* **52**, 4823–4828 (2013).
7. Yu, K., et al. The formation mechanism of binary semiconductor nanomaterials: shared by single-source and dual-source precursor approaches. *Angew. Chem. Int. Ed.* **52**, 11034–11039 (2013).
8. Yu, K., et al. Mechanistic Study of the role of primary amines in precursor conversions to semiconductor nanocrystals at low temperature. *Angew. Chem. Int. Ed.* **53**, 6898–6904 (2014).
9. Zhang, H., Hyun, B. R., Wise, F. W. & Robinson, R. D. A generic method for rational scalable synthesis of monodisperse metal sulfide nanocrystals. *Nano Lett.* **12**, 5856–5860 (2012).
10. Nevers, D. R., et al. Mesophase formation stabilizes high-purity magic-sized clusters. *J. Am. Chem. Soc.* **140**, 3652–3662 (2018).
11. Wang, L. et al. Precursor self-assembly identified as a general pathway for colloidal semiconductor magic-size clusters. *Adv. Sci.* **5**, 1800623 (2018).
12. Haynes W. M. CRC Handbook of Chemistry and Physics, 97th ed. CRC Press 2017.
13. Ithurria S. & Dubertret B. Quasi 2D colloidal CdSe platelets with thicknesses controlled at the atomic level. *J. Am. Chem. Soc.* **130**, 16504–16505 (2008).
